# Genomics, Exometabolomics, and Metabolic Probing Reveal Conserved Proteolytic Metabolism of *Thermoflexus hugenholtzii* and Three Candidate Species From China and Japan

**DOI:** 10.3389/fmicb.2021.632731

**Published:** 2021-05-03

**Authors:** Scott C. Thomas, Devon Payne, Kevin O. Tamadonfar, Cale O. Seymour, Jian-Yu Jiao, Senthil K. Murugapiran, Dengxun Lai, Rebecca Lau, Benjamin P. Bowen, Leslie P. Silva, Katherine B. Louie, Marcel Huntemann, Alicia Clum, Alex Spunde, Manoj Pillay, Krishnaveni Palaniappan, Neha Varghese, Natalia Mikhailova, I-Min Chen, Dimitrios Stamatis, T. B. K. Reddy, Ronan O’Malley, Chris Daum, Nicole Shapiro, Natalia Ivanova, Nikos C. Kyrpides, Tanja Woyke, Emiley Eloe-Fadrosh, Trinity L. Hamilton, Paul Dijkstra, Jeremy A. Dodsworth, Trent R. Northen, Wen-Jun Li, Brian P. Hedlund

**Affiliations:** ^1^School of Life Sciences, University of Nevada, Las Vegas, Las Vegas, NV, United States; ^2^School of Life Sciences, Sun Yat-sen University, Guangzhou, China; ^3^State Key Laboratory of Biocontrol, Guangdong Provincial Key Laboratory of Plant Resources and Southern Marine Science and Engineering Guangdong Laboratory, Zhuhai, China; ^4^Department of Plant and Microbial Biology, The BioTechnology Institute, University of Minnesota, St. Paul, MN, United States; ^5^The Department of Energy Joint Genome Institute, Berkeley, CA, United States; ^6^Environmental Genomics and Systems Biology Division, Lawrence Berkeley National Laboratory, Berkeley, CA, United States; ^7^Department of Biological Sciences, Center of Ecosystem Science and Society, Northern Arizona University, Flagstaff, AZ, United States; ^8^Department of Biology, California State University, San Bernardino, CA, United States; ^9^Nevada Institute of Personalized Medicine, University of Nevada, Las Vegas, Las Vegas, NV, United States

**Keywords:** exometabolomics, thermophile, genomics, *Chloroflexi*, *Thermoflexus*, *Thermoflexus hugenholtzii*, metagenome-assembled genomes

## Abstract

*Thermoflexus hugenholtzii* JAD2^T^, the only cultured representative of the *Chloroflexota* order *Thermoflexales*, is abundant in Great Boiling Spring (GBS), NV, United States, and close relatives inhabit geothermal systems globally. However, no defined medium exists for *T. hugenholtzii* JAD2^T^ and no single carbon source is known to support its growth, leaving key knowledge gaps in its metabolism and nutritional needs. Here, we report comparative genomic analysis of the draft genome of *T. hugenholtzii* JAD2^T^ and eight closely related metagenome-assembled genomes (MAGs) from geothermal sites in China, Japan, and the United States, representing “*Candidatus* Thermoflexus japonica,” “*Candidatus* Thermoflexus tengchongensis,” and “*Candidatus* Thermoflexus sinensis.” Genomics was integrated with targeted exometabolomics and ^13^C metabolic probing of *T. hugenholtzii*. The *Thermoflexus* genomes each code for complete central carbon metabolic pathways and an unusually high abundance and diversity of peptidases, particularly Metallo- and Serine peptidase families, along with ABC transporters for peptides and some amino acids. The *T. hugenholtzii* JAD2^T^ exometabolome provided evidence of extracellular proteolytic activity based on the accumulation of free amino acids. However, several neutral and polar amino acids appear not to be utilized, based on their accumulation in the medium and the lack of annotated transporters. Adenine and adenosine were scavenged, and thymine and nicotinic acid were released, suggesting interdependency with other organisms *in situ*. Metabolic probing of *T. hugenholtzii* JAD2^T^ using ^13^C-labeled compounds provided evidence of oxidation of glucose, pyruvate, cysteine, and citrate, and functioning glycolytic, tricarboxylic acid (TCA), and oxidative pentose-phosphate pathways (PPPs). However, differential use of position-specific ^13^C-labeled compounds showed that glycolysis and the TCA cycle were uncoupled. Thus, despite the high abundance of *Thermoflexus* in sediments of some geothermal systems, they appear to be highly focused on chemoorganotrophy, particularly protein degradation, and may interact extensively with other microorganisms *in situ*.

## Introduction

The bacterial phylum *Chloroflexota* (synonym *Chloroflexi*) continues to be expanded, revealing a global distribution containing broad phylogenetic and physiological diversity. Currently, isolates capable of anoxygenic photosynthesis, obligate organohalide respiration, autotrophy, chemolithotrophy, carboxydotrophy, and fermentation have been described (Moe et al., 2009; Ward et al., 2018; Islam et al., 2019), and recent reports implicate their importance in thermophilic nitrification (Sorokin et al., 2012; Spieck et al., 2019). According to List of Prokaryotic Names with Standing in Nomenclature (Parte et al., 2020), nine classes of *Chloroflexota* have been validly named; however, the Genome Taxonomy Database (Parks et al., 2018) lists 11 classes, only four of which are represented by axenic cultures. *Chloroflexota* are found in freshwater, marine, and hypersaline environments, contaminated groundwater, and terrestrial geothermal springs, among other habitats (Moe et al., 2009; Krzmarzick et al., 2012; Cole et al., 2013; Dodsworth et al., 2014; Hanada, 2014; Denef et al., 2016; Gomez-Saez et al., 2017; Bayer et al., 2018; Kato et al., 2018; Mehrshad et al., 2018; Ward et al., 2018; Thiel et al., 2019; Kochetkova et al., 2020). However, our knowledge of the physiology and ecology of *Chloroflexota* is far from complete, as exemplified by the high abundance and diversity of marine *Chloroflexota* in the poorly understood class *Dehalococcoidia* and the uncultivated SAR202 cluster (Lloyd et al., 2018; Mehrshad et al., 2018).

Many members of the *Chloroflexota* are difficult to isolate and grow in the laboratory, making detailed physiological investigations challenging, even when an isolate is obtained (Yamada et al., 2006, 2007; Bowman et al., 2013; Dodsworth et al., 2014). Several require or are stimulated by complex organic mixtures (e.g., yeast extract, peptone, environmental extracts) (Yamada et al., 2006, 2007; Löffler et al., 2013; Dodsworth et al., 2014). The lack of a defined medium makes one of the most basic biological questions, “what does it eat?”, difficult to answer. Slow growth, low growth yield, and the common filamentous morphology of *Chloroflexota* can make quantification of growth challenging, furthering difficulties associated with describing physiological characteristics (Bowman et al., 2013; Dodsworth et al., 2014). New approaches are needed to cultivate and characterize hard-to-grow and yet-to-be isolated microorganisms, including many *Chloroflexota*, to better address their physiology and ecology.

*Thermoflexus hugenholtzii* JAD2^T^ was isolated from high-temperature (∼80°C) sediments in Great Boiling Spring (GBS), Nevada, United States, where it can be one of the most abundant organisms (estimated 3.2–60% relative abundance) (Costa et al., 2009; Cole et al., 2013; Dodsworth et al., 2014; Thomas et al., 2019). Similar 16S rRNA gene sequences have been recovered from terrestrial geothermal environments around the world (Engel et al., 2013; Hou et al., 2013; Kato et al., 2018), ranging from 63 to 85°C at circumneutral pH, where they can be abundant [e.g., >8% of 16S rRNA gene sequences (Hou et al., 2013)]. The abundance of *T. hugenholtzii* and close relatives in these springs suggests they contribute significantly to biogeochemical cycling in these systems. Yet, little is known about their metabolic capabilities. Axenic cultures of *T. hugenholtzii* remain difficult to study due to low growth yields (<1 mg dry cell mass L^–1^), filamentous morphology (up to ∼500 μm long), lack of a defined medium, and dependence on complex organic extracts from GBS water for optimal growth. Furthermore, in culture, *T. hugenholtzii* may have the narrowest growth temperature range of any bacterium or archaeon known (67.5–75°C) (Dodsworth et al., 2014).

The genomic revolution has provided a plethora of information regarding the potential activities of microorganisms, yet there is a need to connect this inferred potential to the actual physiology of the organisms. Better linking genomes to phenomes stands to advance our understanding of microorganisms and microbial communities by going beyond genetic surveys and providing evidence of precise functions and critical links between genetic potential and ecosystem function. To gain an understanding of the activity of microorganisms, one needs to look at the consequences of enzymatic action, in conjunction with genomic, transcriptomic, or proteomic information, which provide predictions of metabolic capability and evidence of expression. The advancement of exometabolomics, the analysis of metabolites found outside the cell, enables large-scale interpretations of the activities of microorganism through their interactions with molecules in the environment (Mapelli et al., 2008; Silva and Northen, 2015). Similarly, the use of stable isotope-labeled organic compounds can provide information on both catabolic and anabolic activity of specific compounds. The use of position-specific ^13^C-labeled compounds (i.e., isotopomers) provides even more information, including activities of specific enzymes and rates of different metabolic pathways (Dijkstra et al., 2011a,b; Leighty and Antoniewicz, 2013).

Here, we combined analysis of the draft genome of *T. hugenholtzii* and closely related metagenome-assembled genomes (MAGs) with a study of the exometabolome and stable isotope probing of *T. hugenholtzii* to better understand its metabolism and potential ecological role and help inform environmental studies in systems where *Thermoflexus* species are abundant.

## Materials and Methods

### Genome Sequencing

Cultivation of *T. hugenholtzii* JAD2^T^ for genome sequencing was described in Dodsworth et al. (2014). The genome project for strain JAD2^T^ was created in the Genomes OnLine Database (Mukherjee et al., 2021) (Go0015989) and genome sequencing, assembly, and annotation performed by the Department of Energy Joint Genome Institute (Berkley, CA, United States) (Huntemann et al., 2015). A summary of the project information associated with MIGS version 2.0 compliance (Field et al., 2008) is provided in Supplementary Table 1. Contigs and reads were deposited in GenBank (FYEK00000000 and SRP054824).

A total of eight MAGs were analyzed for comparison to the *T. hugenholtzii* JAD2^T^ genome (Tables 1, 2 and Supplementary Table 2). Sample information and sequencing, assembly, and binning information for GBS85_2, GBS70_5, GBS60_20, and GXS_4 can be obtained from the Integrated Microbial Genomes and Microbiomes system (IMG/M, Chen et al., 2020) (300020145, 3300020139, 3300020153, and 3300000865, respectively), and for HR22 from Kato et al. (2018) and under Bioproject ID PRJDB6348.

**TABLE 1:** *Thermoflexus hugenholtzii* JAD2^T^ genome statistics^a^.

Attribute	Value	% of total
Genome size (bp)	3,216,964	100.00
DNA coding (bp)	2,875,571	89.39
DNA G+C (bp)	2,166,171	67.34
DNA scaffolds	78	100
Total genes	2,997	100
Protein coding genes	2,944	98.23
RNA genes	53	1.77
Pseudo genes	0	0
Genes in internal clusters	427	14.25
Genes with function prediction	2,319	77.38
Genes assigned to COGs	1,928	64.33
Genes with Pfam domains	2,396	79.95
Genes with signal peptides	111	3.70
Genes with transmembrane helices	798	26.63
CRISPR repeats	6	

*^a^Genome statistics obtained from JGI IMG (taxon ID 2140918011).*

**TABLE 2:** Genome information and source for *Thermoflexus hugenholtzii* JAD2^T^ and MAGs^a^.

Name	Length (mbp)	# contigs	N50 contigs	% complete	% contam^b^	Source^c^
*T. hugenholtzii* JAD2^T^	3.22	87	139933	97.27	0.91	Isolated from GBS sediment, United States
*T. hugenholtzii* GBS85_2	3.90	355	15878	94.26	1.82	85°C GBS sediment, United States
*T. hugenholtzii* GBS70_5	2.83	256	15285	87.27	1.82	70°C GBS sediment, United States
*T. hugenholtzii* GBS60_20	2.89	324	11092	86.36	3.82	60°C GBS sediment, United States
“*Candidatus* T. sinensis QQ28”	3.50	558	22079	90.91	0.91	68°C QQ sediment, China
“*Candidatus* T. sinensis GXS_4”	3.01	362	10284	77.27	2.42	74°C GXS sediment, China
“*Candidatus* T. sinensis JZ2_71”	3.03	503	7572	85.91	2.36	63°C JZ sediment, China
“*Candidatus* T. tengchongensis QQ20”	3.96	112	80288	95.45	1.82	68°C QQ sediment, China
“*Candidatus* T. japonica HR22”	2.93	175	34479	90.45	1.09	70°C Bioreactor, Japan

*^a^See Supplementary Table 2 for additional MAG statistics.*

*^b^% contam., % contamination.*

*^c^GBS, Great Boiling Spring; QQ, Qiao Quan (also called Qiaobianrequan); GXS, Gongxiaoshe: JZ, Jinze [see Hedlund et al. (2012, 2015), Hou et al. (2013), and Thomas et al. (2019) for site locations and details for MAGs from GBS and China].*

For MAGs JZ2_71, QQ_20, and QQ_28, raw reads were filtered as in Hua et al. (2015) and high-quality reads were assembled using SPAdes (v3.9.0) (Bankevich et al., 2012). Scaffolds were generated with BBMap (v38.85^1^). Scaffolds with a length > 2.5 kbp were assigned to genomic bins using MetaBAT, which is based on read abundance and tetranucleotide word frequency (Kang et al., 2015). Gene calling was performed using Prodigal (Hyatt et al., 2010). Additional site information for JZ2_71, QQ_20, and QQ_28 can be found under GOLD Biosample IDs Gb0159120 (JZ2) and Gb0187827 (QQ) and in Hedlund et al. (2012).

All MAGs were checked for contamination and completeness using the CheckM (v1.0.11) lineage workflow (Parks et al., 2015). Ribosomal RNA presence and copy number was predicted using metaxa2 (v2.2) (16S, 23S) (Bengtsson-Palme et al., 2015, 2016), and RNAmmer (v1.2) (16S, 23S, 5S) (Lagesen et al., 2007). Transfer RNA count was predicted using tRNAscan-SE (v2.0.2) (Lowe and Eddy, 1997). MIMAG quality (Bowers et al., 2017) determination was made for each MAG based on these results. All genomes were run through GTDB-Tk (v0.1.1) for taxonomic assignment and identification of protein-coding genes (Hyatt et al., 2010; Matsen et al., 2010; Eddy, 2011; Jain et al., 2017; Parks et al., 2018).

For genome-based phylogenetic analysis, 120 ubiquitous single-copy protein-coding genes (i.e., bac120) from *Thermoflexus* genomes were identified and aligned using the Genome Taxonomy Database Toolkit (GTDB-tk) (Parks et al., 2018). These sequences were combined with a selection of other *Chloroflexota* with species-level assignments in GTDB release 86 along with a single *Escherichia coli* K-12 MG1655 marker alignment as an outgroup. GCF_900187885.1 was omitted from the alignment because this genome is duplicated as IMG 2140918011. IQ-Tree (v1.6.7.a) (Nguyen et al., 2014) was used to construct a phylogenomic tree from the produced alignment. Ultrafast bootstrap (Hoang et al., 2017) and SH-like alrt (Nguyen et al., 2014) values as implemented in IQ-Tree were used at 1,000 replicates for each to assess support for nodes of the tree.

### Evaluation of Metabolic Potential

The IMG/M system (Chen et al., 2020), in combination with MAPLE, BlastKOALA, and selected searches and manual curation, was utilized to evaluate the *T. hugenholtzii* genome. Protein sequences were obtained from IMG (IMG Taxon ID: 2140918011) or from NCBI for MAGs and were submitted to MAPLE (Metabolic and Physiological potentiaL Evaluator, v2.3.1) (Takami et al., 2012, 2016; Arai et al., 2018) to determine Kyoto Encyclopedia of Genes and Genomes (KEGG) functional module completion ratios (MCRs) based on the presence or absence of KEGG orthology groups (KOs) (Takami et al., 2012), using the NCBI BLAST search engine with the bi-directional best hit annotation method for KO assignment, using all organisms in the KEGG database. MAPLE automatically assigns KOs to query genes using the KEGG automatic-annotation server (KAAS), maps the assigned KOs to KEGG functional modules, then calculates MCRs based on the presence of KOs within each functional module. MAPLE also assigns a *Q*-value to each MCR, to aid in the predication of functionally operable metabolic pathways based on the presence or absence of genes in a genome. *Q*-values provide a statistical measure of the likelihood that the module was identified by chance, as many modules share KOs and thus MCR should not be interpreted alone (see Takami et al., 2012, 2016). Each MCR, using the whole community category, was evaluated with a *Q*-value < 0.5 considered biologically feasible, meaning the presence of associated genes in an individual genome suggest that the metabolic pathway/biochemistry (i.e., the KEGG module) is capable of functioning^2^.

Genes coding for peptidases and peptidase inhibitors and peptidase genomic abundance comparison to other organisms was done using the MEROPS database batch Blast (v10) (Rawlings et al., 2014, 2018). MAGs and *T. hugenholtzii* protein sequences also were analyzed using the MEROPS database (v12) using BLASTP (NCBI BLAST v2.5.0+) and the merops_scan.lib library for comparison between MAGs and *T. hugenholtzii*. An E-value cutoff of 1E-10 was used and the maximum target sequences matched was set to one.

*Thermoflexus hugenholtzii* proteins were also submitted to BlastKOALA (v2.1) (Kanehisa et al., 2016) to populate KEGG maps for exploring metabolic potential, and the SignalP server (v5.0) (Nielsen et al., 1997; Armenteros et al., 2019) and SecretomeP server (v2.0) (Bendtsen et al., 2005) for identification of predicted signal peptide sequences.

For evaluating the presence of gene clusters encoding predicted nitrous oxide reductase systems (*nos*DYLZ) and aerobic carbon monoxide dehydrogenase systems (*coxMSLF*) in MAGs, BLAST+ (Camacho et al., 2009) in the web-based Galaxy platform (Cock et al., 2015) was performed using the translated *T. hugenholtzii* JAD2^T^ genes (*nos*DYLZ, 2413742816-18 and 2143742820; *coxMSLF*, 2143740265-68, 2143742206-09) as the query. Individual amino acid databases for protein-coding genes for MAGs were created and the *T. hugenholtzii* JAD2^T^ sequences were queried using Megablast (Zhang et al., 2000; Morgulis et al., 2008) against each metagenomic bin database. The top hit in each MAG was carefully examined to assess the quality of the annotation, as reported in Supplementary Table 5 and described in results.

### Cultivation of *Thermoflexus hugenholtzii* JAD2^T^ for Exometabolomics

*Thermoflexus hugenholtzii* was cultivated and metabolites were identified in the medium before and after cultivation to determine substrates and products of growth. The cultivation medium was prepared according to the enrichment medium used in Dodsworth et al. (2014) containing the complex carbon sources peptone and yeast extract, except that peptone was increased to 1.0 g/L. Briefly, 20 mL of GBS salts medium, prepared anaerobically, was distributed to 165 mL serum bottles and pressurized with 1 atm of overpressure of N_2_. After autoclaving and relieving the excess pressure, peptone, phosphate buffer, and vitamin solutions were added anaerobically just before inoculation. Filter-sterilized air was added to each bottle for a final concentration of 1% O_2_. An exponential-phase inoculum was added at 1/100 vol/vol. Control replicates were treated identically but received a sham inoculation of 1/100 vol/vol sterile medium. The bottles were returned to 1 atmosphere over pressure with N_2_. Sterile controls, representing the starting medium, were stored at 4°C in the dark for 7 days (*n* = 4). The inoculated replicates (*n* = 5) and another set of sterile controls (*n* = 5) were incubated in the dark with no shaking at ∼74 (±2)°C for 7 days.

After 7 days of incubation, all samples were placed on ice and then centrifuged at 18,514 rcf for 30 min at 4°C in sterile 50 mL Falcon tubes. The supernatant was decanted and filter-sterilized using 0.2 μm PES filters (VWR) into sterile 50 mL Falcon tubes. Filters were rinsed by the passage of 20 mL of sterile nanopure water prior to filtering the supernatant to remove potential chemical contaminants. Supernatants were stored at −80°C and shipped to Joint Genome Institute (JGI) on dry ice for high-performance liquid chromatography tandem mass spectrometry (HPLC–MS/MS) analysis. All samples were processed together.

Cell density was determined by concentrating 1.0 mL of culture from each replicate for 10 min at 22,442 rcf at 4°C. The supernatant was decanted, and the cell pellet was re-suspended in 200 μL of nanopure water. 5.0 μL of the concentrated subsample was loaded onto a Petroff-Hausser counter (#3900; Hausser Scientific Partnership) and photographed using an Olympus BX51 phase-contrast microscope fitted with a V-TV1x-2 camera (Olympus). Measurements of filament length and density were used to determine cell numbers using an average individual cell length of 4.0 μM (Dodsworth et al., 2014).

### Exometabolomics Measurements by HPLC–MS/MS

HPLC–MS/MS was used to identify metabolites. 1 mL media samples with or without *T. hugenholtzii* growth were desalted and extracted using solid-phase extraction cartridges (Bond Elut PPL, 6 mL, 500 mg, #12255001, Agilent). Each cartridge was pre-equilibrated with 1 mL methanol (MeOH) (3×), then 1 mL H_2_O (5x), then all water expelled with air. Each sample was then acidified with HCl by adding 20 μL of 6 M HCl to 1 mL media, briefly vortexing, then flowing through the PPL cartridge. Each cartridge was then rinsed with 1 mL of 0.01 M HCl (2×) and air-dried. Eluent was collected following rinses of each cartridge with 1 mL MeOH (2×) and 1 mL acetonitrile (2×) into a 5 mL Eppendorf tube. Eluent extracts of the desalted media were then dried in a SpeedVac (SPD111V, Thermo Scientific) and stored at −80°C.

In preparation for HPLC-MS/MS analysis, dried extracts (eluents) were resuspended in 110 μL MeOH with internal standards (2-Amino-3-bromo-5-methylbenzoic acid, 1 μg mL^–1^, #R435902; d4-lysine, 10 μg mL^–1^, #61619210; d5-benzoic acid, 10 μg mL^–1^, #217158 – Sigma), centrifuge-filtered through a 0.22 μm hydrophilic PVDF membrane (UFC40GV0S, Millipore), and placed into HPLC vials. HPLC-MS/MS was performed on extracts using an Agilent 1290 LC stack, with MS and MS/MS data collected using a Q Exactive Orbitrap MS (Thermo Scientific, San Jose, CA, United States) in centroid format in both positive and negative ion mode. Full MS spectra were collected from *m/z* 70–1,050 at 70,000 resolution, with MS/MS fragmentation data acquired using stepped 10, 20, and 30 eV collision energies at 17,500 resolution. Source settings of the mass spectrometer included a sheath gas flow rate of 55 (au), auxiliary gas flow of 20 (au), sweep gas flow of 2 (au), spray voltage of 3 kV and capillary temperature of 400°C. Between each sample injection, a blank was run consisting of 100% methanol. Normal-phase chromatography was performed using a ZIC-pHILIC column (Millipore SeQuant ZIC-pHILIC, 150 × 2.1 mm, 5 μm, polymeric), at 40°C, at a flow rate of 0.25 mL^−1^ with a 2 μL injection volume for each sample. The HILIC column was equilibrated with 100% buffer B (90:10 ACN:H_2_O w/5 mM ammonium acetate) for 1.5 min, diluting buffer B down to 50% with buffer A (H_2_O w/5 mM ammonium acetate) for 23.5 min, down to 40% B over 3.2 min, to 0% B over 6.8 min, and followed by isocratic elution in 100% buffer A for 3 min. Metabolites were identified based on exact mass and retention time and comparing MS/MS fragmentation spectra to purchased standards. Raw data files can be obtained through the JGI genome portal under project name “Hedlund 2017 exometabolomics of *Thermoflexus hugenholtzii* JAD2,” Project ID: 1196374^3^.

HPLC-MS/MS data were analyzed using a custom Python code (Yao et al., 2015). Metabolite identification was performed by comparing detected *m/z*, retention time and MS/MS spectra from experimental data to that of compound standards run using the same LC–MS methods. A positive identification was given when retention time and *m/z* matched that of the standard. For peaks that had associated MS/MS, the highest level of positive identification was achieved when the spectra matched that of the standard. This information is summarized in Supplementary Table 6.

HPLC-MS/MS peak-height values for compounds identified in each treatment were compared to determine biological activity, thermal degradation, or thermal production. The treatment with *T. hugenholtzii* growth for 7 days was compared to incubated abiotic controls to determine biological activity, while the sterile starting medium was compared with incubated abiotic controls to determine abiotic effects of high-temperature incubation. Each metabolite was classified according to the Human Metabolome Database (HMDB) hierarchical classification system (Wishart et al., 2018), to simplify links between the LC–MS/MS-identified compounds and genomic data. Metabolites were deemed to have been significantly consumed/degraded or produced if they passed all of the following criteria: (i) at least two of the three treatments’ peak height values were normally distributed according to a Shapiro-Wilk test (*p* > 0.05); (ii) either of the comparisons between the non-incubated treatment and the incubated control treatment or the incubated control and the culture treatment showed significant differences when subjected to a Tukey’s HSD test (*p* < 0.05); and (iii) at least one treatment had a mean peak height intensity (au) > 10^5^. If samples did not match *m/z* and retention times for standards, then they were removed from analysis. If significant compounds were found to have a peak height below 10^5^ in all treatments, then they were removed from analysis. If significant compounds were found to have a peak height at or below 10^5^ in some but not all treatments, then individual chromatograms were manually inspected. Compounds were excluded from analysis if satisfactory peak shape was not found upon manual inspection. Shapiro-Wilk and Tukey’s HSD tests were performed using R version 3.4.3.

### Cultivation of *Thermoflexus hugenholtzii* JAD2^T^ for ^13^C-Labeled Substrate Metabolic Probing

^13^C-labeled metabolic probing was conducted with both position-specific and uniformly labeled substrates, and oxidation of the labeled carbon was assessed by using an isotope spectrometer. The cultivation medium used was based on Dodsworth et al. (2014) and was similar to the exometabolomics medium described above but was scaled up to accommodate multiple head space gas samples (see Supplementary Table 7). An exponential-phase inoculum was added to triplicate bottles at 1/100 vol/vol, 15 mL of pure CO_2_ was added to provide enough CO_2_ (300–2,000 μmol mol^–1^) for subsequent ^13^C-CO_2_ analysis (see below), and then cultures were incubated at 75°C for the duration of the experiment.

At 98.75 h of growth (early exponential phase), position-specific ^13^C-labeled substrates or uniformly ^13^C tricarboxylic acid (TCA) metabolites or amino acids were administered to cultures in the peptone-based medium described above. Each ^13^C treatment was performed in triplicate. ^13^C position-specific substrate additions consisted of filter-sterilized solutions (21.4 μmol substrate-C mL^–1^) of sodium pyruvate (1-^13^C and 2,3-^13^C), sodium acetate (1-^13^C and 2-^13^C), and glucose (1-^13^C and uniformly (U) ^13^C-labeled) (99 atom fraction %; Cambridge Isotope Laboratories, Andover, MA, United States). Uniformly ^13^C-labeled substrate additions consisted of citrate, L-serine, L-cysteine, L-alanine, and succinate (99 atom fraction %; Cambridge Isotope Laboratories, Andover, MA, United States) at a final concentration of 4.0 μg mL^–1^. ^13^C-CO_2_ production rate controls were given natural abundance (i.e., non-^13^C-enriched) pyruvate, acetate, and glucose (as described above for the ^13^C-labeled compounds). A time 0-h headspace sample (10 mL) was taken immediately prior to ^13^C-labeled substrate additions, and 1–2 headspace samples (10 mL each) were taken per 24-h period for the next ∼180 h. Cooling was minimized during sampling by placing bottles in a pre-heated (75°C) water bath.

The 10 mL headspace samples were injected into a Tedlar air-sample bag (Zefon International, Ocala, FL, United States) and increased in volume by diluting with CO_2_-free air after injecting samples. This was done to facilitate a sample run time of ∼10 min. on a Picarro 2101-*i* CO_2_ and CH_4_ isotope spectrometer (Picarro Inc., Sunnyvale, CA, United States). Picarro data were recorded as 30-s averages of δ^13^CO_2_ over a period of near-constant delta readings.

Cultures for monitoring the rate of CO_2_ production were set up as described above but without ^13^C-labeled compounds. Headspace samples (10 mL) were taken over the duration of the experiment and run on a LICOR 6262 (Licor Inc., Lincoln, NE, United States) to determine headspace CO_2_ concentrations.

Triplicate compound stability controls were also performed. For the sterile compound stability tests, 20 mL of GBS salts medium, prepared anaerobically, was distributed into 165 mL serum bottles and prepared essentially as described above except that no inoculum or additional CO_2_ were added. These controls were incubated for ∼180 h at 75°C to mimic the conditions of the ^13^C-labeled compound additions in the larger Wheaton bottle cultures (see Supplementary Text 1). One final sample (∼30 mL) was taken for analysis on the Picarro as described above.

To evaluate the stability of added compounds at growth temperatures, the ^13^CO_2_ production rate from *T. hugenholtzii* cultures was compared with that of sterile controls by converting the volume of CO_2_ present to moles of CO_2_ using the ideal gas law and calculating the ^13^C atom fraction. Using the calculated atom fraction values, we applied a mass balance equation for isotope mixing to determine the contribution of ^13^C-CO_2_ from biotic and abiotic degradation processes (see Supplementary Text 1 and Supplementary Table 3 for additional information).

### Data Availability

All genomic data are available on one or more data servers, as summarized in Supplementary Table 2. Raw metabolomics data are available on IMG through the genome portal under project name “Hedlund 2017 exometabolomics of *Thermoflexus hugenholtzii* JAD2,” Project ID: 1196374^3^.

## Results

### *Thermoflexus hugenholtzii* JAD2^T^ Genome Overview

The *T. hugenholtzii* JAD2^T^ draft genome is 3,216,964 bp in size and consists of 78 scaffolds (size range, 121–4,05,611 bp), with a G + C content of 67.34%. The genome encodes 2,997 predicted genes, of which 2,944 are protein-coding (89.39%). Also annotated are 48 tRNA-encoding genes, a single copy of 5S and 16S rRNA genes, and a fragmented 23S rRNA gene. A total of 1,928 genes (64.33%) and 1,141 genes (38.07%) were assigned to COGs and KO groups, respectively (Supplementary Table 4). Additional details concerning the genome and interpretations can be found in Table 1 and Supplemental Information.

**FIGURE 1:**
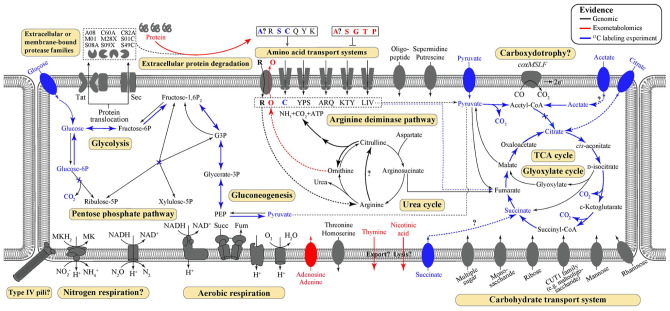
Predicted and demonstrated metabolic features of *Thermoflexus hugenholtzii* JAD2^T^. Genomic predictions are shown in black/gray. Evidence derived from exometabolomics experiments and ^13^C-labeling experiments are shown in red and blue, respectively. Experimental evidence was also supported by genomic data, except for thymine and nicotinic acid secretion. Extracellular or membrane-bound protease families refer to MEROPS protease families. Amino acid transport systems show single letter amino acid codes or strings thereof. Succ, succinate; Fum, fumarate; PEP, phosphoenolpyruvate; MK, menaquinone; G3P, glycerol-3-phosphate.

Key metabolic features of *T. hugenholtzii* were predicted from the genome (Figure 1) based on IMG/M annotations, BlastKOALA, and selected manual annotations, and pathways were evaluated for feasibility based on MAPLE MCRs, where *Q*-values below 0.5 were considered feasible (Takami et al., 2012, 2016; Arai et al., 2018). Most central carbon metabolic pathways [e.g., glycolysis, TCA cycle, pentose-phosphate pathway (PPP), gluconeogenesis] are feasible, except for the Entner-Doudoroff pathway (Supplementary Table 5). Notably, a gene encoding an archaeal-type fructose 1,6-bisphosphatase aldolase/phosphatase (K01622) (Say and Fuchs, 2010) (Supplementary Table 6), involved in gluconeogenesis, was found. In addition, transporters for carbohydrates, arabinogalactan oligomer/maltooligosaccharide, monosaccharides, multiple sugars, rhamnose, and ribose were identified (Supplementary Table 5). Transporters for thiamine (IMG gene ID# 2143740997 and 2143740999) and ascorbate [phosphotransferase system (PTS), 2143742986–2143742988] were also identified (Supplementary Table 5). Genes coding for nucleoside ABC transporters and a putative hydroxymethylpyrimidine transporter were found (Supplementary Table 5), along with those encoding numerous ABC type-II transporters (Supplementary Table 5).

While genes coding for a nitrous oxide reductase system (2413742816-18 and 2143742820, Supplementary Data Sheet 1) and a dissimilatory nitrite reduction to ammonium system (*nrfHA* 2143740544 and 2143740545) were identified manually, the MAPLE analysis provided no support for any complete nitrogen or sulfur metabolism modules, including nitrogen fixation (M00175), ammonia oxidation (M00528), complete denitrification (M00529), dissimilatory nitrate reduction to ammonium (M00530), assimilatory nitrate/nitrite reduction (M00531), complete nitrification (i.e., comammox; M00804), assimilatory sulfate reduction (M00176), dissimilatory sulfate reduction (M00596), or respiratory thiosulfate oxidation (SOX pathway, M00595) (Supplementary Table 5). The urea cycle (M00029) (Supplementary Table 5) appears feasible via a bifunctional carbamate kinase (EC2.7.2.2), fulfilling the role of a carbamoyl-phosphate synthase (EC6.3.4.16) (Supplementary Table 5). No components for nitrate/nitrite transport (M00438) or sulfate transport systems (M00185) were found, although all components for a NitT/TauT family transport system (M00188), involved in sulfonate/nitrate/taurine transport, were present (Supplementary Table 5). A full aerobic type-I *coxMSLF* was identified (2143740265-68 and 2143742206-09) (Supplementary Data Sheet 1). Genes coding for NADH:quinone oxidoreductase, succinate dehydrogenase, cytochrome c oxidase, and an F-type ATPase lacking a prototypical delta subunit were also identified (Supplementary Table 4), which is typical of some other *Chloroflexota* (Takami et al., 2012; Chadwick et al., 2018). Components for neither photosystem (M00597 and M00598), nor the 3-hydroxypropionate autotrophic pathway (Supplementary Table 5) were detected, and no other autotrophic pathways were encoded in the genome. KEGG modules for the synthesis of bacteriochlorophylls, carotenoids, and rhodopsins were largely unpopulated, and manual searches failed to reveal homologs of key genes for these biosynthetic pathways.

### Predicted Protein and Amino Acid Metabolism

**FIGURE 2:**
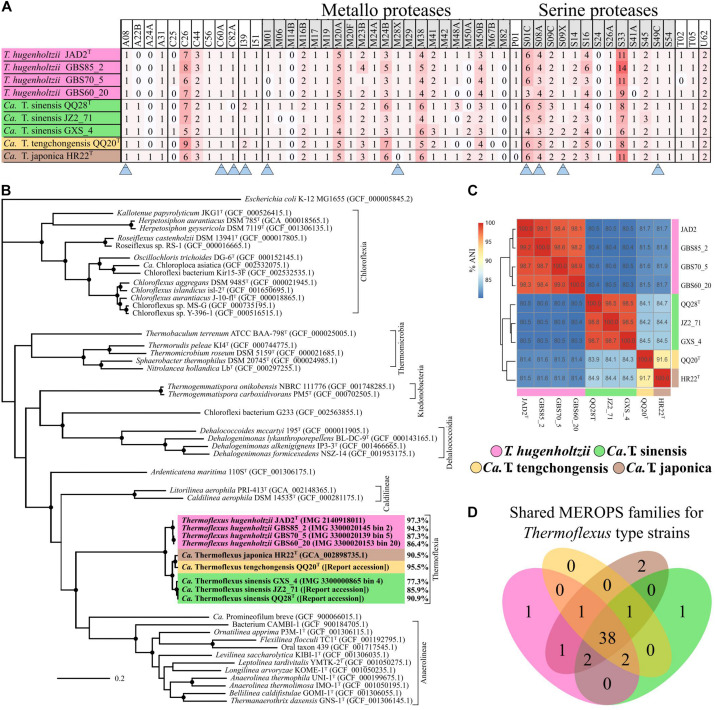
*Thermoflexus* evolutionary relationships and abundance and conservation of MEROPS families. **(A)** Heatmap for the presence and abundance of MEROPS families in all MAGs and *T. hugenholtzii* JAD2^T^, with families containing predicted extracellular or lipid-anchored proteases marked (arrows). **(B)** Phylogenomic bac120 tree for the phylum *Chloroflexota.* The alignments for all *Chloroflexota* GTDB genomes with classification at the species level and *E. coli* K-12 MG1655 (outgroup) were used to generate the phylogenomic tree, black circles indicate bootstrap value ≥ 95% (1,000 replicates). Percentages associated with *T. hugenholtzii* JAD2^T^ and *Thermoflexus* MAGs represent estimated genome completeness. **(C)** Average nucleotide identity (ANI) matrix for all MAGs and *T. hugenholtzii* JAD2^T^. **(D)** Venn diagram for MEROPS families shared between *T. hugenholtzii* JAD2^T^ and MAGs representing type material for “*Candidatus* Thermoflexus” species.

*Thermoflexus hugenholtzii* JAD2^T^ contains an unusually high abundance and diversity of annotated peptidases, with 133 genes coding for peptidases and five peptidase inhibitors (Figure 2A, Table 3 and Supplementary Table 5). 4.4% of total genes coded for members of peptidase families, placing *T. hugenholtzii* JAD2^T^ in the top 3.6% of *Bacteria* and *Archaea* for the percentage of genes belonging to a MEROPS protein family^4^. 17 of the annotated endo- and exopeptidases are predicted to be membrane-bound or extracellular (Supplementary Table 5). Secretion of these proteases would be feasible through the Sec pathway (SecD/F, SecGYA, YidC, Ftsy, and Ffh) and the twin-arginine translocation system (TatAC), along with family I and II signal peptidases (LepB and LspA) (Supplementary Table 5). The most abundant protease families are the Metallo (M) and Serine (S) peptidases, with 51 and 53 genes, respectively.

**TABLE 3:** MEROPS statistics for *Thermoflexus hugenholtzii* JAD2^T^ ^a^.

MEROPS members of peptidase families	133 (not including 5 inhibitors)
% of total genes coding for members of peptidase families	4.44%
Most abundant families	Metallo (51) and Serine (53) peptidases
Bacteria and Archaea with ≥ 133 members*	13.7%
Bacteria with ≥ 133 members*	14.1%
Bacteria and Archaea with ≥ 4.44% of total genes coding for members of peptidase families*	3.6%
Bacteria with ≥ 4.44% of total genes coding for members of peptidase families*	3.7%
Endopeptidase count	52 (39.10% of total)
Exo-, amino-, carboxy-, di- peptidase count	56 (42.11% of total)

*^a^Data generated using MEROPS version 10 and comparison to Peptidases in Whole Genome Sequences (^∗^), https://www.ebi.ac.uk/merops/cgi-bin/compgen_index?type=P, accessed 02/11/2019. Six erroneous organism entries were removed.*

Predicted family M01 (2143742583) and M28F (2143740571), both aminopeptidases, have lipoprotein signal peptides (Supplementary Table 5). M28F has been shown to result in free arginine, lysine, and leucine (Fundoiano-Hershcovitz et al., 2005). Other genes coding for M-family exopeptidases or peptidases that result in small peptide fragments or free amino acids were predicted to be cytoplasmic and might be important for processing transported oligopeptides or protein turnover (e.g., M01, M03B, M16, M17, M19, M24, M28, M29, M32, M42, and M79). For example, one M19 dipeptidase (2143740867) is predicted to generate free glycine and eight family M20 exopeptidases are predicted to generate free amino acids.

Nine genes belong to the S1 family of Serine proteases, including seven members of the S1C family. This family of endopeptidases resides in the periplasm of Gram-negative bacteria and can also serve as a general molecular chaperone (Krojer et al., 2002) (Supplementary Table 5). Five of these seven genes contained lipoprotein signal peptides (214374072, 2143741777, and 2143742813), Sec signal peptides (2143740725), or non-classical secretion sequences (2143742057) (Supplementary Table 5). Four peptidases belonged to family S8A, subtilisin endopeptidases with broad substrate specificity, with three containing lipoprotein signal peptides (2143741277) or non-classical secretion sequences (2143740323 and 2143742883) (Supplementary Table 5). A gene (2143742360) belonging to family S9B prolyl endopeptidases contained a Sec signal peptide (Supplementary Table 5). Family S33, aminopeptidases that preferentially cleave proline from peptides, contained 18 genes, but no secretion sequences were identified. Seven genes encoded family C26 peptidases, consisting of gamma-glutamyl hydrolases closely linked to pyrimidine biosynthesis, arginine biosynthesis, and the urea cycle. Genes for inhibitors belonging to families I39 (2), a broad inhibitor of endopeptidases, I51 (1), an inhibitor of serine carboxypeptidases, and I87 (2), an inhibitor of FtsH, were found (Supplementary Table 5).

Several ABC transporters might enable the transport of oligopeptides, free amino acids, or other protein degradation products. Genes for ABC transporters for branched-chain amino acids (LivKHGFM), oligopeptides (OppABDCF), and spermidine/putrescine (PolDGBA) were present (Supplementary Table 5). A particularly large gene cluster (IMG gene IDs 2143741899-2143741889) coding for peptide/nickel, polar amino acid, branched-chain amino acid, and hydrophobic amino acid transporters was identified (Supplementary Table 5), along with a putative glutamine transport system (2143742870-2143742872; Supplementary Table 5). A gene for an amino acid/polyamine/organocation transporter (2143740310), along with two genes for ornithine carbamoyltransferases (2143743137 and 2143741572), and one for a carbamate kinase (2143741573), were found (Supplementary Table 5). These genes are part of the arginine deiminase pathway that is responsible for the import and catabolic use of arginine and the export of ornithine.

In contrast, no ABC transporters for general L-amino acids (AapJQMP), cysteine (TcyABC and TcyJKLMN), lysine (LysX1X2Y), histidine (HisJMQP), glutamine (GlnHPQ), arginine (ArtJIMQP), hydroxyproline (LhpPMNO), D-methionine (MetQIN) arginine/ornithine (AotJMQP), glutamate/aspartate (GltIKJL), arginine/lysine/histidine/glutamate (BgtBA), arginine/lysine/histidine (ArtPQR), lysine/arginine/ornithine/histidine/octopine (PA5152-55), neutral amino acids/histidine (NatBCDAE), dipeptide/heme/ δ-aminolevulinic acid (DppABCDF), or dipeptide (DppEBCD) transport were found (Supplementary Table 5).

Several pathways were identified for the catabolic use of amino acids or interconversion of amino acids, which are likely important for the proteolytic lifestyle of *Thermoflexus*. For example, homoserine, threonine, and glycine could potentially be converted into pyruvate from serine (EC4.3.1.19) and aspartate could be degraded to fumarate (EC6.3.4.4, 4.3.2.2; 6.3.4.5, 4.3.2.2), feeding central carbon metabolism (Supplementary Table 5). Arginine, glutamate, or glutamine could also be broken down to 2-oxoglutarate, feeding the TCA cycle, suggesting they may be important substrates for *T. hugenholtzii* JAD2^T^. Alternatively, using TCA cycle-derived 2-oxoglutarate or arginine, glutamate, or glutamine as substrates, ornithine, citrulline, and proline biosynthesis appears possible (Supplementary Table 5). Serine and isoleucine biosynthesis from aspartate by way of homoserine, threonine, and glycine appeared possible (M00018 and M00570) (Supplementary Table 5) and suggests these may also be important substrates. Cysteine biosynthesis from serine (M00021) or homocysteine and serine (M00338) was not feasible according to MAPLE (Supplementary Table 5) and no other routes for biosynthesis were observed (Supplementary Table 5), suggesting *Thermoflexus* might be auxotrophic for cysteine. *T. hugenholtzii* JAD2^T^ was also predicted to be incapable of *de novo* synthesis of asparagine, aspartate, cysteine, glycine, histidine, homocysteine, homoserine, isoleucine, lysine, methionine, phenylalanine, phosphoserine, serine, threonine, and tyrosine, suggesting amino acid scavenging and/or interconversion might be critical to *Thermoflexus* (see Supplementary Text 1 for additional information).

### Environmental Distribution and Metabolic Potential of *Thermoflexus* MAGs

Eight *Thermoflexus* MAGs were identified in public databases, including four high-quality MAGs (GBS85_2, QQ20, QQ28, and HR22) and four medium-quality MAGs (GBS70_5, GBS60_20, and GXS_4, JZ2_71) (Figure 2B and Supplementary Table 2). All bins contained one copy of each rRNA gene, except JZ2_71, which lacked a 5S rRNA, presumably due to genome incompleteness or a binning problem (Supplementary Table 2). Some rRNA genes were fragmented across different contigs. These MAGs derived from four different sediment samples within GBS, several springs in the Tengchong region of southwest China (Qiao Quan spring, Gongxiaoshe spring, and Jinze pool), and an enrichment culture derived from a subsurface gold mine in Japan. These thermal environments range from 60 to 85°C and pH from 6.7 to 7.3, which is generally consistent with the very narrow range for laboratory growth of *T. hugenholtzii* [67.5–75°C; pH 6.5–7.75 (Dodsworth et al., 2014)]. The reason for the high relative abundance of *T. hugenholtzii* in GBS sediments above maximum growth temperature in the laboratory is unknown (Cole et al., 2013; Thomas et al., 2019). Additional information about these springs is provided elsewhere (Hedlund et al., 2012; Hou et al., 2013; Peacock et al., 2013; Kato et al., 2018; Thomas et al., 2019).

A phylogenetic analysis using the bac120 gene set showed that all MAGs formed a deep-branching monophyletic group within the phylum *Chloroflexota*, with *T. hugenholtzii* JAD2^T^ being the only cultured representative (Figure 2B). The phylogenetic analysis and average nucleotide identity (ANI) values showed that the genomes included four species-level groups. MAGs GBS85_2, GBS70-5, and GBS60_20 belonged to *T. hugenholtzii* (Figure 2C). GXS_4, JZ2_71, and QQ28^T^ belonged to a species cluster sharing 98.5–98.8% ANI, herein called “*Candidatus* Thermoflexus sinensis.” HR22^T^ and QQ20^T^ formed a cluster but shared only 91.7% ANI and were therefore designated “*Candidatus* Thermoflexus japonica” and “*Candidatus* Thermoflexus tengchongensis,” respectively.

All MAGs showed similar MCRs and were generally consistent with the metabolic potential of *T. hugenholtzii* JAD2^T^ (Supplementary Table 5). *T. hugenholtzii* JAD2^T^ was most similar to the con-specific high-quality MAG GBS85_2, with only ∼4.6% of modules having differing MCRs. With a few exceptions, MCRs for carbohydrate metabolism modules were similar across MAGs and mirrored *T. hughenholtzii* JAD2^T^. However, “*Candidatus* Thermoflexus tengchongensis QQ20^T^” did not encode the full gene complement for glycolysis (M00001 and M00002) or gluconeogenesis (M00003) modules. The non-oxidative PPP (M00007) was not feasible in “*Candidatus* Thermoflexus japonica HR22^T^.” The glyoxylate cycle (M00012) was only feasible in the *T. hugenholtzii* group and “*Candidatus* Thermoflexus tengchongensis QQ20^T^.” All MAGs lacked the delta subunit of the F-type ATPase. The aerobic type-I *coxMSLF* was conserved across all MAGs. However, *Ca*. T. sinensis GXS_4 and “*Candidatus* T. japonica HR22^T^” had notably lower sequence similarity for *cox*MLF, and GBS60_20 had lower sequence similarity for *coxM*, with respect to *T. hugenholtzii* JAD2^T^ (Supplementary Table 3). The NreB-NreC (dissimilatory nitrate/nitrite reduction) two-component regulatory system (M00483) was only feasible in *T. hugenholtzii* JAD2^T^, GBS85_2, and GBS60_20. A full nitrous oxide reductase system (*nosZDYL*) was found in all MAGs except for *Ca*. T. sinensis JZ2_71, which was missing *nosL*, and GBS60_20, which was missing *nosYL* and had a notably lower sequence similarity for *nosZD*, with respect to *T. hugenholtzii* JAD2^T^ (Supplementary Table 5). In the case of noted absences, this may be due to incomplete genomes from metagenome binning rather than true genomic absences.

Comparison of MEROPS families between MAGs and *T. hugenholtzii* JAD2^T^ revealed a total of 50 MEROPS protease families and two inhibitor families (Figure 2D and Supplementary Table 5). Of these, 46 protease families and the two inhibitor families (I39 and I51) were represented by at least one gene in every MAG and *T. hugenholtzii* JAD2^T^ (Figure 2D and Supplementary Table 5). MAGs “*Candidatus* T. sinensis QQ28^T^,” “*Candidatus* T. tengchongensis QQ20^T^,” “*Candidatus* Thermoflexus japonica HR22^T^,” and *T. hugenholtzii* JAD2^T^ shared 38 families, with only one family (M82) unique to *T. hugenholtzii* JAD2^T^, one family (M14B) unique to “*Candidatus* T. sinensis QQ28^T^,” and two families (A24A, S24) unique to “*Candidatus* T. japonica HR22^T^” (Figure 2D). Within the *T. hugenholtzii* group, 39 families were shared by all members (Supplementary Data Sheet 2). Within the “*Candidatus* T. sinensis” group, 41 families were shared by all members (Supplementary Data Sheet 2). M and S families were dominant across all MAGs and *T. hugenholtzii* JAD2^T^, with 14 and 10 unique families found in all genomes, respectively (Figure 2A). Families C26, M20A, M38, S01C, S08A, and S33 were the most abundant families (average count: 7, 5, 5, 5, 4, and 9, respectively) (Figure 2A and Supplementary Table 5). These numbers are likely an under-estimation for some less complete MAGs.

With a few exceptions, nucleotide and amino acid metabolism modules were similar across all MAGs and mirrored *T. hugenholtzii* JAD2^T^ (Supplementary Table 5). However, inosine monophosphate biosynthesis (M00048) was not feasible in any of the “*Candidatus* T. sinensis” MAGs or the medium-quality GBS70_5 MAG, while it was in all others (Supplementary Table 5). Similarly, MAGs generally possessed the same transporters as *T. hugenholtzii* JAD2^T^ (Supplementary Table 5). A molybdate transport system (M00189) was not feasible in the *T. hugenholtzii* group or *Candidatus* T. tengchongensis QQ20^T^ but was in all others. A ribose transport system (M00212) was not feasible in any of the “*Candidatus* T. sinensis” MAGs; an inositol-phosphate transport system (M00599) was feasible in “*Candidatus* T. japonica HR22^T^” and “*Candidatus* T. tengchongensis GXS_4” and “QQ28^T^,” but not in any others or *T. hugenholtzii* JAD2^T^. A PTS transporter for ascorbate was found in all MAGs and *T. hugenholtzii* JAD2^T^.

### Exometabolomics

**FIGURE 3:**
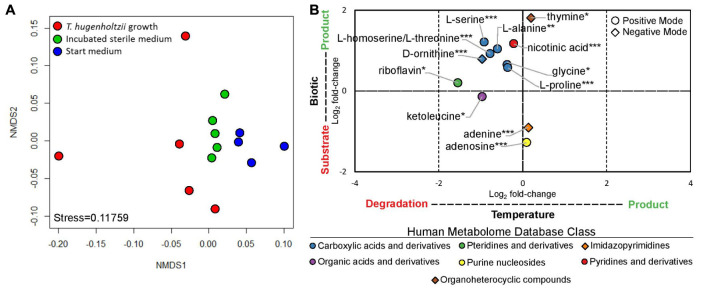
Exometabolomic profile for *Thermoflexus hugenholtzii* JAD2^T^. **(A)** NMDS plot and **(B)** Log_2_ fold-changes in peak height for statistically significant results. NMDS plots were generated from positive and negative mode peak heights of compounds. Each dot represents a single sample. Replicate #1, with the most growth, is the left-most red data point. Log_2_ fold-changes in peak height for statistically significant results were determined by ANOVA and *post hoc* Tukey Honest Significant Differences between treatments for each compound (Shapiro-Wilk test used to determine normality). Data were considered significant if either treatment comparison yielded a *p*-value < 0.05 for the Tukey HSD. HPLC–MS/MS positive mode (circle) or negative mode (diamond) peak height data were used for all compounds. If a compound was found significant in both positive and negative mode, positive mode data only are presented. Color indicates the Human Metabolome Database (HMDB) metabolite Class for specific compounds. Compound identification, 1, glycine; 2, alanine; 3 homoserine/threonine; 4, proline; 5, serine; 6, monomethyl glutaric acid; 7, ketoleucine; 8, riboflavin; 9, adenosine; 10, nicotinic acid; 11, ornithine; 12, adenine; 13, thymine. *s indicate confidence in compound identification (***, HPLC–MS/MS data matches a fragmented in-house standard; **, *m/z* and retention time match in-house standard but MS/MS fragmentation is difficult to interpret; *, *m/z* and retention time match in-house standard but no fragmentation data are available; if samples did not match *m/z* and retention times for standards, then they were removed from analysis).

*Thermoflexus hugenholtzii* JAD2^T^ grew well (0.26–4.6 × 10^7^ cells/mL) in cultures for exometabolomics, resulting in an average cell yield of 1.1 × 10^7^ cells/mL (Supplementary Text 1). NMDS plots showed the exometabolomic profile representing *T. hugenholtzii* growth to have much higher variability than the sterile medium and sterile incubated controls (Figure 3A and Supplementary Table 6), demonstrating the difficulty to reproducibly grow this organism and limiting our ability to identify statistically significant differences in the abundance of substrates and products. Nevertheless, thirteen compounds that significantly increased or decreased in abundance due to biological or thermal activity were identified with high confidence (Figures 1, 3B and Supplementary Table 6). Only two compounds contained in the medium were substrates for *T. hugenholtzii* JAD2^T^ and were represented by the HMDB classes *Imidazopyrimidines* (adenine) and *Purine nucleosides* (adenosine). Compounds produced due to biological activity were largely represented by the HMDB class *Carboxylic acids and derivatives* (6), with one representative of *Pyridines and derivatives* (nicotinic acid), and one representative of *Organoheterocyclic compounds* (thymine). All compounds of the *Carboxylic acids and derivatives* class belonged to the sub-class *Amino acids*, *peptides*, and *analogs*, with direct parent compounds of alpha-amino acids (glycine) or L-alpha amino acids (L-alanine, L-homoserine/L-threonine, L-proline, L-serine, and L-ornithine). L-homoserine and L-threonine were not distinguishable with HPLC–MS/MS. In addition, many di- and tri-peptides were identified as possible biological products but were not confirmed with purified standards (“Untargeted metabolomics”; see metabolomics data availability above).

In addition, thermal degradation of ketoleucine (4-methyl-2-oxovaleric acid) and riboflavin, belonging to the HMDB classes *Organic acids and derivatives* and *Pteridines and derivatives*, respectively, were observed.

### ^13^C Metabolic Probing

**FIGURE 4:**
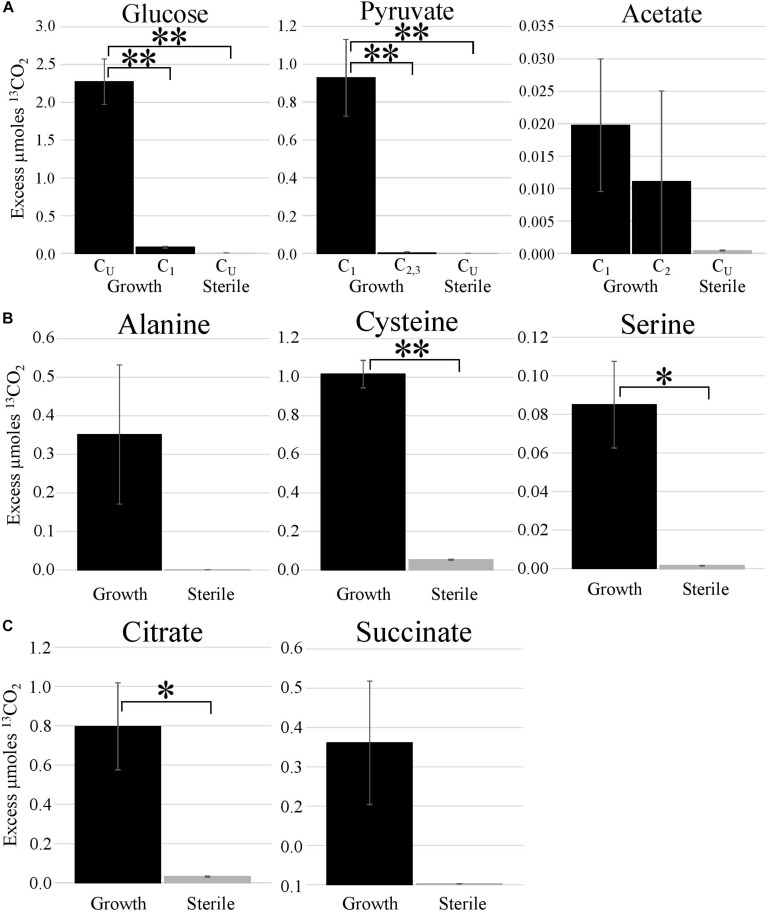
Metabolic activities demonstrated by stable isotope experiments. Excess μmoles of ^13^CO_2_ produced from ^13^C-labeled substrates by *T. hugenholtzii* JAD2^T^ and sterile controls. Isotopomers of glucose, pyruvate, and acetate [**(A)**; CU, uniformly ^13^C-labeled]; uniformly ^13^C-labeled amino acids **(B)**; uniformly ^13^C-labeled TCA metabolites **(C)**. **(A)** *s, indicate statistically different, ANOVA, *post hoc* Tukey HSD. *** <0.005. **(B,C)** *s, indicate statistically different, Student’s *t*-test (two-tailed, unequal variance), ** <0.05, * <0.10.

Metabolic probing of *T. hugenholtzii* JAD2^T^ with ^13^C-labeled compounds demonstrated heterotrophic activity on a variety of organic substrates, including glucose, organic acids, amino acids, and TCA-cycle intermediates (Figures 1, 4). ^13^CO_2_ was recovered from both universally ^13^C-labeled and ^13^C_1_-labeled glucose, the latter providing evidence for the oxidative PPP, albeit at a low rate relative to glucose oxidation through glycolysis. ^13^C_1_-labeled pyruvate was oxidized to ^13^CO_2_, providing evidence of pyruvate decarboxylase at the transition between glycolysis and the TCA cycle; however, no ^13^CO_2_ was recovered from ^13^C_2,3_-labeled pyruvate, demonstrating an uncoupling of glycolysis and the TCA cycle (Figures 1, 4), suggesting acetate might be sequestered through the glyoxylate cycle or possibly for fatty acid biosynthesis (Figures 1, 4). ^13^CO_2_ was produced from the universally ^13^C-labeled amino acids cysteine and serine, and the TCA-cycle intermediate citrate. Alanine and succinate also may have been oxidized to ^13^CO_2_, although those data were not statistically significant.

## Discussion

### Protein and Amino Acid Metabolism

*Thermoflexus hugenholtzii* JAD2^T^ only grows well in the laboratory on a complex medium containing peptone as a carbon, nitrogen, and energy source, suggesting peptides and amino acids sustain *T. hugenholtzii* growth. However, no growth on casamino acids or multiple single amino acids has been observed (Dodsworth et al., 2014). Here, we combined genomic and phenomic analyses to demonstrate that *T. hugenholtzii* JAD2^T^ does indeed digest extracellular peptides and that some free amino acids are transported and oxidized, whereas others accumulate in the extracellular *milieu*. In all, 17 of the 133 annotated peptidases in *T. hugenholtzii* JAD2^T^ were predicted to be extracellular or lipid-anchored (Figures 1, 2A, Table 3, and Supplementary Table 5). *Thermoflexus* MAGs from several geothermal springs in China and Japan showed a similar repertoire of proteases (Figure 2D, Supplementary Table 5, and Supplementary Data Sheet 2), suggesting a conserved proteolytic lifestyle for the genus.

The extracellular accumulation of alanine, glycine, homoserine/threonine, ketoleucine, ornithine, proline, and serine in culture supernatants was consistent with the lack of substrate-specific, general amino acid, and neutral amino acid transporters. However, the ^13^C metabolic probing experiments did provide evidence that serine and possibly alanine can be metabolized. It is possible that these amino acids are taken up by other transport systems, albeit at a low rate and/or affinity. The lack of annotated general and neutral amino acid transporters, along with more specific amino acid transporters, was surprising given the protease repertoire of *T. hugenholtzii* JAD2^T^. Conversely, no branched-chain, hydrophobic, or charged amino acids accumulated, which is consistent with an abundance of branched-chain and hydrophobic amino acid transporters in the genome (Supplementary Table 5).

No amino acids decreased in abundance in the presence of *T. hugenholtzii* growth. This result suggests that individual amino acids were liberated from extracellular peptides at a rate similar to or less than uptake by *T. hugenholtzii*. Thus, the balance of extracellular peptidase activity may be finely tuned with amino acid uptake in *T. hugenholtzii*. Potentially, this helps *T. hugenholtzii* in the natural environment by ensuring energy and biomass conserved in extracellular proteases is not wasted on amino acid production beyond cellular demand. This would also reduce the free amino acid pool in the extracellular environment and reduce competition.

Extracellular homoserine/threonine and proline accumulation was consistent with the presence of three genes coding for threonine/homoserine efflux transporters (RhtA). This may be indicative of a mechanism for balancing intracellular metabolite pools to facilitate the reactions of central carbon metabolic pathways when feeding on proteins (Livshits et al., 2003). In support of this hypothesis, all amino acids that accumulated in the medium are genomically predicted to be utilized in biosynthetic and catabolic pathways (Supplementary Table 5). Serine has also been shown to inhibit threonine and isoleucine biosynthesis (Hama et al., 1991), further suggesting metabolic inhibition may be taking place. Metabolic inhibition may contribute to the low cell density observed in *T. hugenholtzii* cultures when grown on peptides as a carbon and energy source but may be relieved *in situ* by cometabolism with neighboring species.

Consumption of amino acids as a primary carbon and energy source would also lead to excess intracellular nitrogen, which would have to be excreted. Genes coding for necessary gamma-glutamyl hydrolases and an alternative enzyme (EC2.7.2.2) enabling the urea cycle to function (Supplementary Table 5) provide such a mechanism. Additionally, gluconeogenesis would be expected under growth on amino acids, which is feasible by an archaeal-type fructose 1,6-bisphosphatase aldolase/phosphatase (Say and Fuchs, 2010). This enzyme may help *T. hugenholtzii* JAD2^T^ metabolize amino acid-derived heat-labile triosephosphates into heat-stable fructose 6-phosphate, rendering metabolite pools stable, and allow metabolic flexibility free from transcriptional regulation (Say and Fuchs, 2010). Malate dehydrogenase (EC1.1.1.40) appears to be responsible for the start of gluconeogenesis through pyruvate formation from amino acids fed into the TCA cycle, rather than a phosphoenolpyruvate carboxykinase, as was confirmed by a manual search for phosphoenolpyruvate carboxykinase.

Ornithine and ketoleucine accumulated in the medium. Ornithine is a by-product of the urea cycle and ketoleucine can be formed from the incomplete decomposition of branched-chain amino acids, both of which were predicted from the genome (i.e., a complete urea cycle and high abundance of branched-chain amino acid transporters). In addition, genes for parts of the arginine deiminase pathway, a pathway for the catabolism of arginine, were identified; however, an arginine deiminase (EC3.5.3.6) was not identified (Zuniga et al., 2002). This pathway results in the import of arginine, export ornithine, and production of ATP. Ornithine accumulation during *T. hugenholtzii* JAD2^T^ growth suggests this pathway may be active despite a gene coding for an arginine deiminase not being identified.

*Thermoflexus* appears to rely on a significant complement of amino acids, which is consistent with an obligately proteolytic lifestyle. For example, many amino acids appear to be metabolic dead ends, meaning that they do not feed into central carbon metabolic pathways, and others have no recognizable *de novo* biosynthetic clusters. For example, isoleucine, leucine, valine, methionine, phenylalanine, and tryptophan could be transported but are possible metabolic dead ends. The *de novo* biosynthesis of several amino acids did not appear possible due to the absence of single genes (i.e., histidine or glutamine, proline, ornithine, arginine, and citrulline or serine). Most of these absences are supported by comparative genomics with *Thermoflexus* MAGs, which suggests these are true absences and not artefacts from incomplete genome assembly. This could mean that these amino acids must be scavenged or interconverted, or alternatively that *Thermoflexus* harbors undefined genes capable of carrying out the missing reactions. In cases where a vast majority of genes are present for a pathway, the presence of undefined genes seems likely (e.g., histidine, tyrosine, phenylalanine, and phosophserine biosynthesis, LysW pathway). Attempts to design a defined medium using a diversity of amino acid mixtures and CCMP metabolites did not support growth. A defined medium would allow further exploration of the capacity for *de novo* amino acid biosynthesis by *T. hugenholtzii* JAD2^T^ and the identification of novel enzymes and metabolic pathways.

### Broad Heterotrophic Activity and Central Carbon Metabolism

The ^13^C metabolic probing experiments demonstrated broad heterotrophic activity of *T. hugenholtzii* JAD2^T^, despite the challenge of growing it in pure culture. This result is generally consistent with genomic predictions, and broad heterotrophic activity demonstrated in GBS sediments where *Thermoflexus* is abundant (Murphy et al., 2013; Thomas et al., 2019). One surprising result is the apparent uncoupling of glycolysis and the TCA cycle, as evidenced by the decarboxylation of ^13^C_1_ of pyruvate but not ^13^C_2,3_ (Figure 4). The *T. hugenholtzii* JAD2^T^ genome contains two annotated pathways for oxidation of C_1_ from pyruvate during formation of acetyl-CoA through pyruvate-ferredoxin oxidoreductase (IMG gene ID# 21437407219-2143740722, 2143741077, 2143741234, and 2143741244–2143741246) or pyruvate dehydrogenase (2143740152 and 2143740153). If the resulting acetyl group were transferred to oxaloacetate by citrate synthase (2143741275), then C_2_ and C_3_ of pyruvate would be oxidized over multiple cycles of the TCA cycle, which was not observed (Figure 4). The absence of this activity suggests acetate, produced from C_2_ and C_3_ of pyruvate, is either excreted or fully sequestered in biomass. Paradoxically, metabolic probing with ^13^C-acetate suggested some acetate may be oxidized to ^13^CO_2_, although the return of ^13^CO_2_ from either of the isotopomers was not statistically significant (Figure 4). By comparison, *Chloroflexus aurantiacus* excretes acetate through an archaeal-type ADP-forming acetyl CoA synthetase (Schmidt and Schönheit, 2013), which is also present in *Thermoflexus* (2143741578). *C. aurantiacus* also assimilates acetate through the glyoxylate cycle when growing mixotrophically with H_2_ and CO_2_ (Zarzycki and Fuchs, 2011). It is possible that *Thermoflexus* has similar reactions with acetate, although more definitive experiments would be needed to probe these ideas.

The very high ratio of ^13^CO_2_ production from universally labeled glucose, compared with ^13^C_1_-glucose (∼25:1) indicates that glycolysis is highly active relative to the oxidative PPP, which would decarboxylate the C_1_ position via 6-phosphogluconate dehydrogenase (2143742524). Interpretation of the ^13^C-glucose and ^13^C-pyruvate data together suggest a ten-fold higher rate of glycolysis relative to the oxidative PPP, since the only ^13^CO_2_ production from universally labeled glucose would occur for C_3_ and C_4_ due to pyruvate-ferredoxin oxidoreductase or pyruvate dehydrogenase. This result might not be surprising given the presence of nucleotides and nucleosides in yeast extract coupled with predicted nucleoside transporters, the demonstrated uptake of adenine and adenosine (Figure 3), and the presence of a ribose transporter in all *Thermoflexus* genomes (Figure 1). A similarly high ratio of ^13^CO_2_ production from universally labeled glucose compared with ^13^C_1_-glucose was seen in 60°C GBS sediments (Thomas et al., 2019). The production of thymine and nicotinic acid are not understood based on incomplete biosynthetic pathways in all *Thermoflexus* genomes and warrants future work.

### Potential Alternative Metabolic Strategies

Both nitrous oxide and nitrite were predicted to serve as terminal electron acceptors for anaerobic growth; however, neither metabolism could be confirmed with *T. hugenholtzii* cultures. A nitrous oxide reductase system was conserved across *Thermoflexus* species except MAG GBS60_20, which was obtained from 60°C sediments. Denitrification is active in GBS (Dodsworth et al., 2011). High rates of N_2_O flux have been measured in the GBS source pool (∼82°C) and to a slightly lesser degree at a high-temperature shelf (∼82°C), with minimal flux observed at low-temperature sites (∼65°C) (Hedlund et al., 2011). N_2_O released by leaky denitrification or other sources at high temperature may provide a terminal electron acceptor for *T. hugenholtzii* strains inhabiting this temperature range. At lower-temperature sites, the source for *T. hugenholtzii* GBS60_20 (Thomas et al., 2019), other organisms may have complete denitrification pathways or outcompete *T. hugenholtzii*, resulting in the loss of the nitrous oxide reductase system in *T. hugenholtzii* adapted to these temperatures. However, to date, no consumption of nitrous oxide has been observed for *T. hugenholtzii* JAD2^T^ cultures under anaerobic conditions (1% or 5% total volume headspace gas; data not shown). Similarly, no stimulation of growth under anaerobic conditions has been observed in the presence of nitrite (2 mM) (Dodsworth et al., 2014), so the function of the encoded dissimilatory nitrite reduction to ammonium system has also not been verified.

Genes coding for a type-I *coxMSLF* are conserved across all *T. hugenholtzii* MAGs, but carboxydotrophy has also not been observed for *T. hugenholtzii* JAD2^T^. It has been suggested that this system may provide a means for *Chloroflexota* to persist in times of low nutrient availability and situations requiring dormancy by providing an alternative energy source (Islam et al., 2019). This system may provide a means for survival for *Thermoflexus* in times of low organic carbon availability, such as a lack of allochthonous C sources. However, no consumption of carbon monoxide (5% of headspace) was observed when *T. hugenholtzii* was grown in the presence of O_2_ (1% of headspace) or anaerobically with nitrite (2 mM) or nitrous oxide (5% of headspace) (data not shown).

### Ecological Implications and Potential Metabolic Interdependencies

From an ecological perspective, it is intriguing that *T. hugenholtzii* JAD2^T^ seems to be an obligate chemoheterotroph that depends on proteins and amino acids in light of the high abundance of this organism and close relatives in some hot spring sediments and non-photosynthetic mats (Cole et al., 2013; Hou et al., 2013). In GBS, *T. hugenholtzii* is an abundant member of the sediment community around 80°C (3.2–60% estimated relative abundance), several meters away from photosynthetic mats, which are well-formed in GBS sediments below ∼70°C (Cole et al., 2013). It seems unlikely that microbially derived, autochthonous proteinaceous substrates would be sufficient to support such an abundant organism. However, it is possible that high rates of phage-mediated microbial community turnover may enable *Thermoflexus* to grow to high abundance based on the use of microbial cell lysates serving as a primary source of proteins and extracellular biomass precursors (Breitbart et al., 2004). Similarly, predatory lifestyles have been reported for other *Chloroflexota* (e.g., *Herpetosiphon* spp.) (Livingstone et al., 2018) and perhaps *Thermoflexus* abundance follows a Lotka-Volterra predator-prey relationship, as the estimated abundance of *Thermoflexus* has been observed to fluctuate within GBS sediments over time (e.g., Cole et al., 2013; Thomas et al., 2019). Future environmental studies concerning *Thermoflexus* may benefit from co-occurrence analyses (Chaffron et al., 2010; Freilich et al., 2010; Barberán et al., 2012). Alternatively, or in conjunction with above, *T. hugenholtzii* may rely on allochthonous proteins, which could be addressed through analysis of the natural abundance stable isotopes. In addition, the presence of multiple carbohydrate and sugar importers and complete CCMPs suggest that *T. hugenholtzii* should be able to utilize these substrates as well, although these substrates do not support growth as sole carbon and energy sources (Dodsworth et al., 2014). In the natural environment, *T. hugenholtzii* and close relatives may serve as important players in the initial breakdown of allochthonous proteins, providing a pool of free amino acids for consumption by other community members. It is common to find plant, insect, and animal remains at the sediment-water interface in geothermal systems, and these biomass sources may serve as important proteinaceous substrates for *Thermoflexus*.

Heterotrophy is widespread within the *Chloroflexota*, including both photosynthetic and non-photosynthetic taxa that are abundant and common in circumneutral to alkaline pH geothermal features in Yellowstone National Park. For example, the genera *Roseiflexus* and *Chloroflexus*, both within the *Chloroflexaceae*, are highly abundant in phototrophic mats in the outflow channels of the Octopus Spring and Mushroom Spring in the Lower Geyser Basin, where *in situ* metabolism has been studied in some detail (e.g., van der Meer et al., 2005, 2007). Although these two genera are capable of autotrophy via the 3-hydroxypropionate pathway, stable-isotope probing experiments have shown them to assimilate both bicarbonate and acetate *in situ*, which is consistent with their preferred mode of photoheterotrophic growth in culture (van der Meer et al., 2010). In these communities, heterotrophic growth is dominant under low light conditions and at night, when most carbon assimilated by *Chloroflexaceae* is derived from fermentation products and other photosynthates released by *Cyanobacteria*.

*Thermoflexus* extends this general heterotrophic lifestyle to higher temperatures within geothermal systems and similarly it is also likely to be interdependent on other microorganisms. Although mixotrophic *Aquificaceae* are present in both GBS and throughout geothermal springs in Tengchong (Dodsworth et al., 2015; Hedlund et al., 2015), they are not abundant in sediments hosting abundant *Thermoflexus*. However, each of these springs have a long water residence time [e.g., 1–2 days for GBS (Costa et al., 2009)] and they do host abundant *Aquificaceae* populations in the overlying water. Thus, it is possible that spatially uncoupled autotroph-heterotroph interactions exist between *Aquificaceae* and *Thermoflexus* that mirror those between photoautotrophic *Cyanobacteria* and *Chloroflexaceae* at lower temperatures. However, in the case of *Thermoflexus*, the metabolic focus might be detrital material or predation rather than direct metabolic coupling. A metabolism focused on detrital proteins or predation is consistent with the requirements of *Thermoflexus* for exogenous proteins, vitamins, cofactors, and unknown compounds present in organic mat extracts for optimal growth. Similarly, amino acids, thymine, and nicotinic acid released by *Thermoflexus* would likely be useful commodities for other community members. These ideas await more incisive experiments to probe these metabolisms in artificial consortia or *in situ*.

## Conclusion

By combining genomic and exometabolomic data, insight into the physiology of *T. hugenholtzii* JAD2^T^ was gained. This synergistic approach allowed us to go beyond genomic predictions, observe the metabolic activity of this minimally culturable organism, and provide confirmation of some, but not all, predictions stemming from genomic analysis. By comparing the *T. hugenholtzii* JAD2^T^ genome to other *Thermoflexus* MAGs, it was further possible to hypothesize that similar yet-to-be cultivated organisms in geothermal environments around the world have comparable metabolic activity and contributions to biogeochemical cycling. These insights into *Thermoflexus* metabolic capabilities provide a new baseline for the continued cultivation effort of this genus and its relative *Chloroflexota*.

### Descriptions of *Candidatus* Species

“*Candidatus* Thermoflexus sinensis” (si’nen.sis) Latin neut. adj. Sinae, Chinese; the Chinese Thermoflexus. The nomenclatural type is the metagenomic bin QQ_bins28 (JAEVEY000000000).

Currently known only from metagenomic sequence data from circumneutral pH geothermal springs in Tengchong, China. Habitat and genomic features suggest a phenotype conforming to the description of the genus *Thermoflexus*. Predicted to be proteolytic, based an abundance of proteases, and facultatively anaerobic, based on cytochrome c oxidase, nitrous oxide reductase, and a dissimilatory nitrite reduction to ammonium system. Possibly carboxydotrophic, based on a type I carbon monoxide dehydrogenase system. In addition to the nomenclatural type, also includes “*Candidatus* T. sinensis” GXS_4 (JAFLMU000000000) and “*Candidatus* T. sinensis” GZ2_71 (JAEVEZ000000000).

“*Candidatus* Thermoflexus tengchongensis” (teng.chong.en’sis) originating from Tenghchong, a region of Yunnan Province, China; the Thermoflexus from Tengchong. The nomenclatural type is the metagenomic bin QQ_bins20 (NCBI ID JAEVEX000000000).

Currently known only from metagenomic sequence data from circumneutral pH geothermal springs in Tengchong, China. Habitat and genomic features suggest a phenotype conforming to the description of the genus *Thermoflexus*. Predicted to be proteolytic, based an abundance of proteases, and facultatively anaerobic, based on cytochrome c oxidase, nitrous oxide reductase, and a dissimilatory nitrite reduction to ammonium system. Possibly carboxydotrophic, based on a type I carbon monoxide dehydrogenase system.

“*Candidatus* Thermoflexus japonica” (ja.pon’i.ca) Latin neut. adj. Japonicus, Japanese; the Japanese Thermoflexus. The nomenclatural type is the metagenomic bin HR22 (BEHY00000000.1).

Currently known only from metagenomic sequence data from an enrichment culture derived from a subsurface gold mine in Japan. Habitat and genomic features suggest a phenotype conforming to the description of the genus *Thermoflexus*. Predicted to be proteolytic, based an abundance of proteases, and facultatively anaerobic, based on cytochrome c oxidase, nitrous oxide reductase, and a dissimilatory nitrite reduction to ammonium system.

## Data Availability Statement

The datasets presented in this study can be found in online repositories. The names of the repository/repositories and accession number(s) can be found in the article/Supplementary Material.

## Author Contributions

MH, AC, AS, MP, KP, NV, NM, I-MC, DS, TR, RO’M, CD, NS, NI, NK, TW, and EE-F sequenced and assembled the genome of *T. hugenholtzii*. J-YJ and W-JL assembled and provided the QQ20, QQ28, and JZ2_71 MAGs. ST, CS, SM, TH, DL, and JD completed the bioinformatic analysis. ST, KT, DP, BH, and PD contributed to the experimental design for ^13^C work. ST, KT, and PD completed the analysis of ^13^C work. ST, KT, and DP carried out all the culture work. ST, KT, BH, LS, BB, and TN contributed to the experimental design for exometabolomic work. RL, LS, BB, KL, and TN performed the exometabolomic analysis. ST, CS, and SM completed the statistical analysis of exometabolomic data. ST interpreted, compiled, and wrote the manuscript with input from all authors.

## Conflict of Interest

The authors declare that the research was conducted in the absence of any commercial or financial relationships that could be construed as a potential conflict of interest.

## Footnotes

1 https://sourceforge.net/projects/bbmap/

2 https://maple.jamstec.go.jp/maple/maple-2.3.1/help.html

3 https://genome.jgi.doe.gov/portal/Hed201tabolomics_FD/Hed201tabolomics_FD.info.html

4 http://merops.sanger.ac.uk/cgi-bin/compgen_index?type=p; accessed on 02/11/2019.
