# Supplementary material for: Genomics, Exometabolomics, and Metabolic Probing Reveal Conserved Proteolytic Metabolism of Thermoflexus hugenholtzii and Three Candidate Species From China and Japan
Source: Front Microbiol. 2021 May 3;12:632731. doi: 10.3389/fmicb.2021.632731 (PMC8129789; doi:10.3389/fmicb.2021.632731)
Supplement: Supplementary file 1 [file Table_1.docx]

Table S1. Project information for genome compliance with MIGS standards for *T. hugenholtzii* JAD2^T^

| **MIGS ID** | **Property** | **Term** |
| --- | --- | --- |
| MIGS 31 | Finishing quality | Level 3: Improved-High-Quality Draft |
| MIGS-28 | Libraries used | Illumina Regular Fragment 300 bp |
| MIGS 29 | Sequencing platforms | Illumina |
| MIGS 31.2 | Fold coverage |  |
| MIGS 30 | Assemblers | Velvet v. 1.1.04 |
| MIGS 32 | Gene calling method | Prodigal 2.5 |
|  | Locus Tag | K362 |
|  | Genbank ID | FYEK00000000 |
|  | GenBank Date of Release | 2017-06-28 |
|  | GOLD ID | Gp0015989 |
|  | BIOPROJECT | PRJNA195829 |
| MIGS 13 | Source Material Identifier | JAD2^T^ |
|  | Project relevance | Biotechnological |
