# Supplementary material for: Genomics, Exometabolomics, and Metabolic Probing Reveal Conserved Proteolytic Metabolism of Thermoflexus hugenholtzii and Three Candidate Species From China and Japan
Source: Front Microbiol. 2021 May 3;12:632731. doi: 10.3389/fmicb.2021.632731 (PMC8129789; doi:10.3389/fmicb.2021.632731)
Supplement: Supplementary file 7 [file Table_7.DOCX]

***Supplementary Material***

1. **Additional information for the cultivation of *T. hugenholtzii* JADT for ^13^C-labeled substrate metabolic probing**

To accommodate multiple headspace gas samples 200 mL of GBS salts medium (1.0g/L peptone), prepared anaerobically, was distributed to 500 mL Wheaton bottles and pressurized with 1 atm of overpressure of N_2_.

Culture bottles were anaerobically prepared at the University of Nevada, Las Vegas, then transported to Northern Arizona University where they were vented to bring the bottles to atmospheric pressure at NAU (0.8 atmospheres). Peptone, phosphate buffer, and vitamin solutions were added anaerobically just before inoculation. Filter-sterilized air was added to each bottle for a final concentration of 1% O_2_.

To compare the ^13^CO_2_ production rate from *T. hugenholtzii* cultures with that of sterile controls, the ideal gas law was used to convert the volume of CO_2_ present in the incubations from various processes and additions, to moles of CO_2_ present from various processes and additions.

PV=nRT

Where n is the number of micromoles of gas; P is pressure in atm; V is volume of the gas in L; T is temperature (°K); and R is the gas constant (0.08205746 (atm*L) / (moles*K)). We also used the atom fraction equation for ^13^C

^13^C atom fraction = ((δ13C/1000+1)*0.011237)/((δ13C/1000+1)*0.011237+1)

to calculate ^13^C atom fraction (x(^13^C)) values from δ^13^C values determined by running samples on the Picarro. Using the calculated atom fraction values, we applied a mass balance equation for isotope mixing to determine the contribution of ^13^C-CO_2_ from different processes and additions in the cultures and abiotic controls.

x(^13^C)_t_V_t_ = x(^13^C)_1_V_1_ + x(^13^C)_2_V_2_ …

Taken together, using cultures with ^13^C-labeled substrate additions, cultures with no ^13^C-substrate additions, cultures used for total CO_2_ production rates, and abiotic ^13^C-CO_2_ controls, we were able to attribute ^13^C-CO_2_ production to either *T. hugenholtzii* metabolism or abiotic processes.

1. **Additional information on inferred metabolic potential for *Thermoflexus hugenholtzii* JAD2^T^**

***Inferred amino acid interconversion and biosynthetic capability***

Ornithine (M00763) and lysine biosynthesis (M00031) appeared possible (Table S4), yet both biosynthesis pathways were missing genes connecting them to the TCA cycle (Table S3). Histidine degradation to glutamate through N-formiminoglutamate appears possible (M00045) (Table S4). The absence of one gene coding for a homoserine acetyltransferase (EC2.3.1.31) may prevent the synthesis of homocysteine and methionine from aspartate or homoserine (Table S3).

Histidine biosynthesis from ribose-5P was missing a single gene coding for an imidazoleglycerol-phosphate dehydratase (EC4.2.1.19) or a histidinol-phosphatase (EC3.1.3.15) but all other enzymes were present (Table S3). There were no noticeable routes for cysteine biosynthesis by *de novo* means or by conversion of methionine or serine due to the absence of a serine O-acetyltransferase (EC2.3.1.30) (Table S3). *De novo* serine and phosphoserine biosynthesis were not possible due to the absence of a single gene (phosphoserine aminotransferase, EC2.6.1.52) yet, other genes necessary for the conversion of glycolysis-derived glycerate-3P to serine were present (EC1.1.1.95, EC3.1.3.3) (Table S3). The absence of EC2.6.1.52 and genes coding for enzymes for the biosynthesis of aspartate from oxaloacetate suggests an inability for *the de novo* biosynthesis of homoserine, threonine, glycine, and isoleucine (Table S3). Tryptophan biosynthesis from D-erythrose 4-phosphate and phosphoenolpyruvate appears possible through the shikimate pathway (M00022, M00023) (Table S3, Table S4). Genes coding for enzymes involved in tryptophan metabolism and lysine degradation were largely absent, suggesting these compounds are not important substrates for *T. hugenholtzii* JAD2^T^ (Table S3). Alanine could be synthesized from or broken down to pyruvate (EC1.4.1.1, EC 2.6.1.44, respectively), but no other pathways for conversion to other amino acids were observed (Table S3). Valine and leucine could be synthesized from pyruvate (TableS3).

***Inferred nucleoside/nucleotide biochemistry***

*T. hugenholtzii* JAD2^T^ appears to be able to degrade and synthesize both adenine and adenosine (Table S3, Table S4). In contrast, neither thymine degradation (M00046) or synthesis (M00053) appear to be possible due to the absence of multiple enzymes (EC2.4.2.6, EC2.4.2.4, EC1.3.1.1, EC1.3.1.2, EC1.17.99.4) (Table S3, Table S4).

1. **Additional information for exometabolomics**

***Accumulation and degradation of other compounds***

Adenine and adenosine were substrates for *T. hugenholtzii* JAD2^T^. This observation was supported by the presence of nucleoside transporters and complete pathways for the degradation of these compounds. Biosynthetic pathways for this compound were also complete in *T. hugenholtzii* and all MAGs, indicating *Thermoflexus* may synthesize them *de novo* when necessary. In contrast, thymine accumulated in the medium, which seems paradoxical given that thymine pathways are incomplete. This was unexpected given that biosynthesis pathways for this compound are incomplete in *T. hugenholtzii* JAD2^T^ and all *Thermoflexus* MAGs.

A slight increase in riboflavin in the presence of growth was observed in the exometabolomic data despite a gene coding for an ECF-type riboflavin transporter, S component being found. More significant is the thermal degradation of riboflavin, which is thought to be stable at higher temperatures over short periods of time. The long incubation times necessary to grow *T. hugenholtzii* JAD2^T^ provide ample opportunity for thermal degradation and production of products, providing deviations in the chemical makeup of the medium over time solely due to temperature. This, along with the demonstrated production and degradation of other compounds due to temperature, highlights the importance of running sterile controls in parallel with cultures during exometabolomic analyses but also illuminates potential challenges when growing thermophiles with long doubling times, due to chemical changes in the medium strictly from temperature.

1. **Cell yields for exometabolomics and an unidentified GBS organic extract**

***An unidentified GBS organic extract stimulated Thermoflexus growth***

The addition of an organic extract derived from Great Boiling Spring, the source of *T. hugenholtzii* JAD2^T^, significantly enhanced growth, suggesting that organic extracts commonly used for microbiological media are limited in some beneficial nutrients. A thiamine transport system was found in *T. hugenholtzii* JAD2^T^, and an ascorbate phosphotransferase system was conserved across the genus, which is consistent with our observations that *T. hugenholtzii* JAD2^T^ is stimulated by high concentrations of vitamins (data not shown). Complete carbohydrate metabolic pathways and a variety of transporters for oligo- and monosaccharides suggest these compounds may be utilized by *T. hugenholtzii* JAD2^T^ and other *Thermoflexus* sp., yet these types of compounds are not capable of serving as sole carbon and energy sources for growth (Dodsworth et al., 2014). These data suggest that yet-to-be-determined key nutrient limitations may contribute to the low cell density observed in *T. hugenholtzii* JAD2^T^ cultures when grown on peptide-based complex media as a carbon and energy source.

|  | cells/mL | Average (cells/mL) |
| --- | --- | --- |
| Exometabolomics |  |  |
| Rep. 1 | 4.6 x10^7^ | 1.1 x10^7^ |
| Rep. 2 | 2.7 x10^6^ |  |
| Rep. 3 | 2.0 x10^6^ |  |
| Rep. 4 | 1.4 x10^6^ |  |
| Rep. 5 | 2.6 x10^6^ |  |
| Organic extract addition | | |
| Rep. 1 | 5.1 x10^7^ | 3.6 x10^7^ |
| Rep. 2 | 3.5 x10^7^ |  |
| Rep. 3 | 1.8 x10^7^ |  |
| Rep. 4 | 4.7 x10^7^ |  |
| Rep. 5 | 3.2 x10^7^ |  |
|  |  |  |

**Cell counts for exometabolomic experiments and hot spring organic extract additions.** Replicate 1 for exometabolomics demonstrated much higher growth than the other replicates within the same treatment.
