# Supplementary material for: Genomics, Exometabolomics, and Metabolic Probing Reveal Conserved Proteolytic Metabolism of Thermoflexus hugenholtzii and Three Candidate Species From China and Japan
Source: Front Microbiol. 2021 May 3;12:632731. doi: 10.3389/fmicb.2021.632731 (PMC8129789; doi:10.3389/fmicb.2021.632731)

Supplemental Figure 1. Genomic arrangement for genes of nitric-oxide reductase (A) and carbon monoxide dehydrogenase (B) in *T. hugenholtzii*.

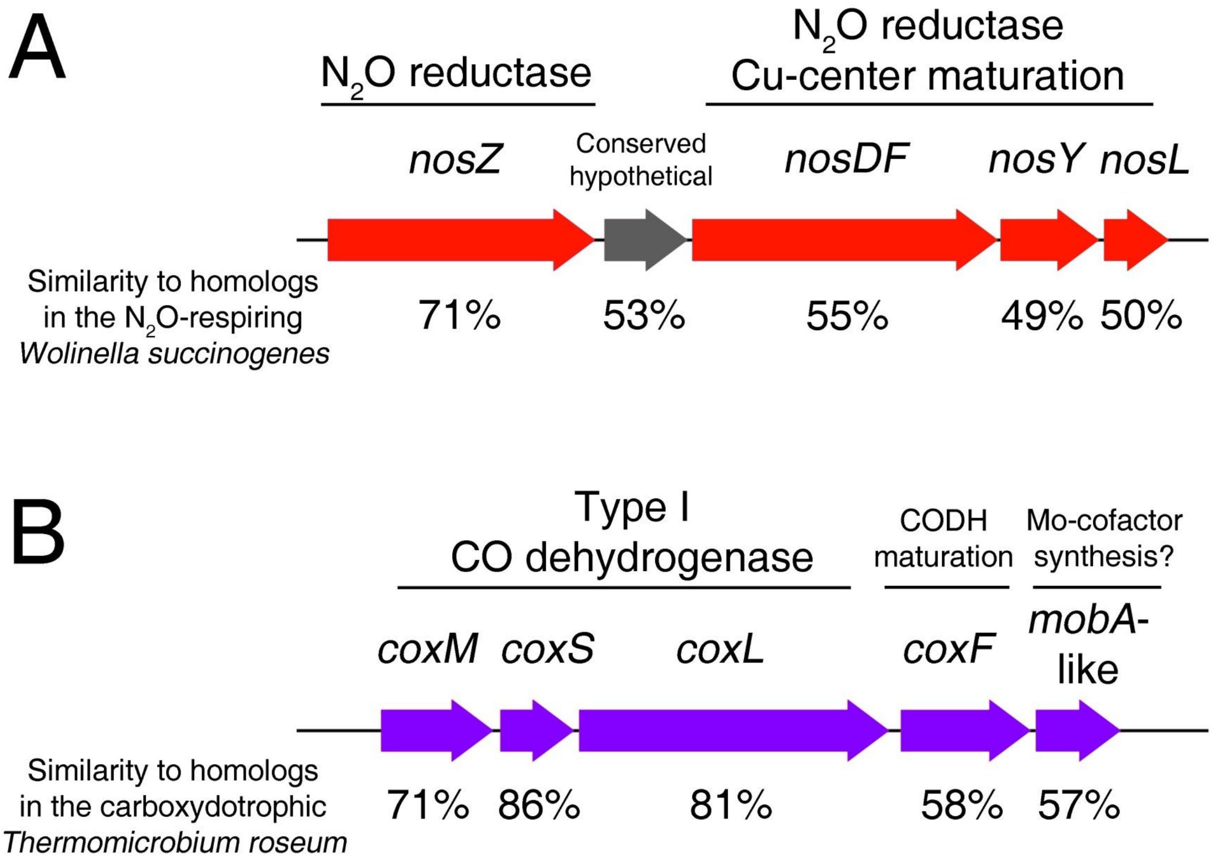

Supplement: Supplementary file 8 [file Data_Sheet_1.PDF]
