# Supplementary figures and images for: Genomics, Exometabolomics, and Metabolic Probing Reveal Conserved Proteolytic Metabolism of Thermoflexus hugenholtzii and Three Candidate Species From China and Japan
Source: Front Microbiol. 2021 May 3;12:632731. doi: 10.3389/fmicb.2021.632731 (PMC8129789; doi:10.3389/fmicb.2021.632731)

Figure S2. Shared MEROPS Families for *T. hugenholtzii* (A) and *Ca. T. sinensis*

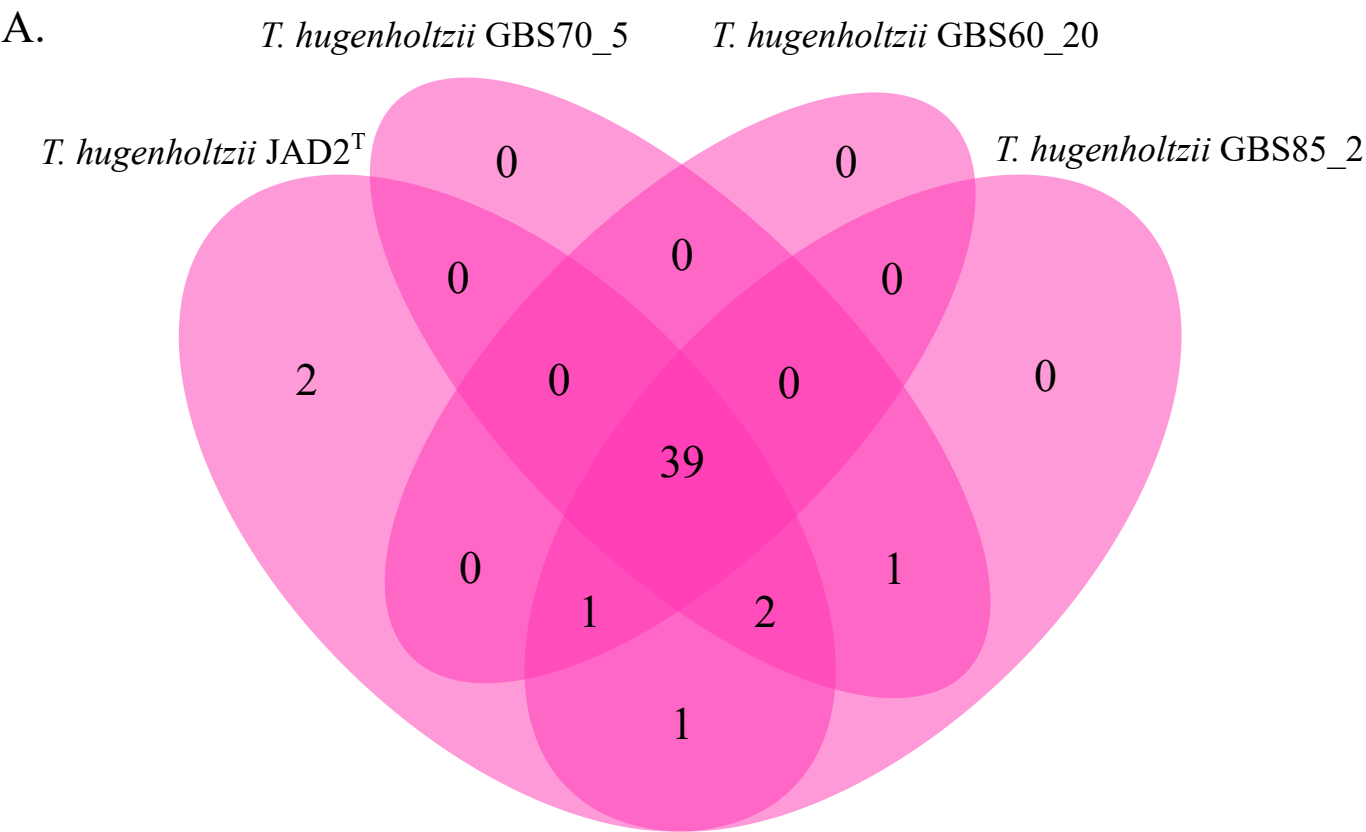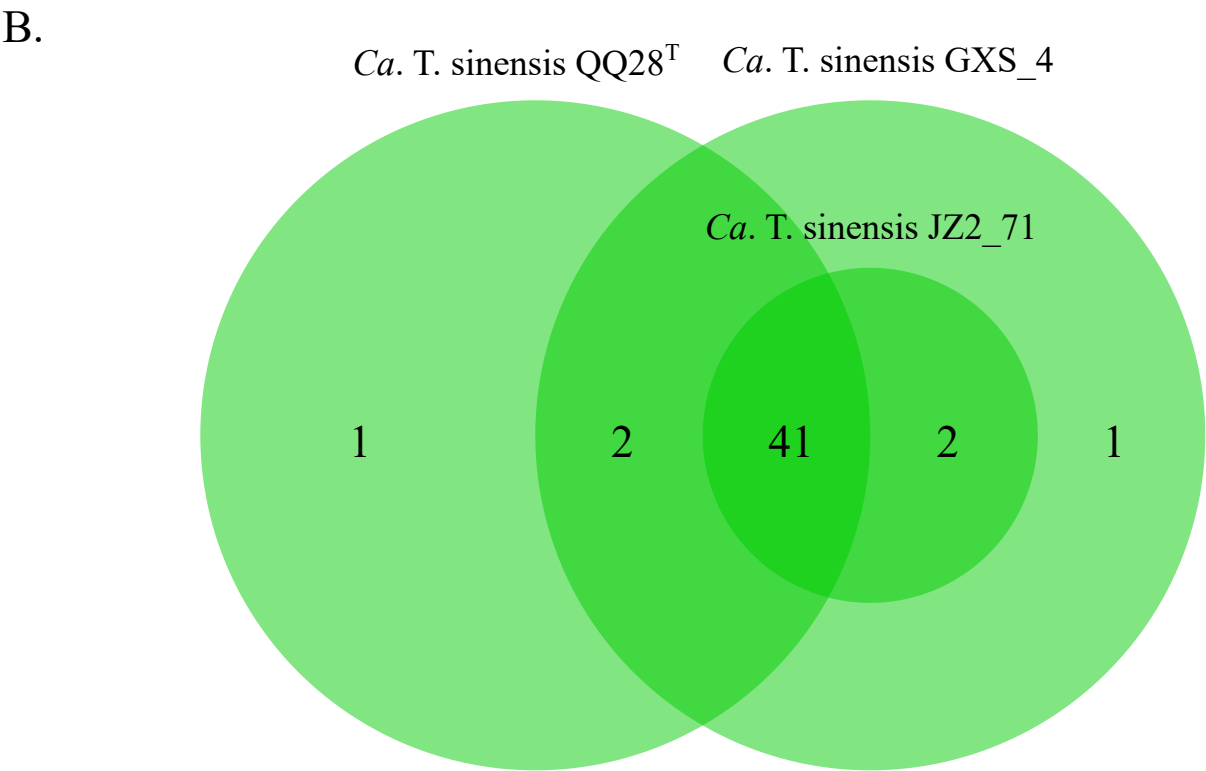

Supplement: Supplementary file 9 [file Data_Sheet_2.PDF]
