# Supplementary material for: Genomics, Exometabolomics, and Metabolic Probing Reveal Conserved Proteolytic Metabolism of Thermoflexus hugenholtzii and Three Candidate Species From China and Japan
Source: Front Microbiol. 2021 May 3;12:632731. doi: 10.3389/fmicb.2021.632731 (PMC8129789; doi:10.3389/fmicb.2021.632731)
Supplement: Supplementary file 10 [file Presentation_1.pdf]

Supplementary Material

1. Additional information for the cultivation of *T. hugenholtzii* JADT for ^{13}C-labeled substrate metabolic probing

To accommodate multiple headspace gas samples 200 mL of GBS salts medium (1.0g/L peptone), prepared anaerobically, was distributed to 500 mL Wheaton bottles and pressurized with 1 atm of overpressure of N_2.

Culture bottles were anaerobically prepared at the University of Nevada, Las Vegas, then transported to Northern Arizona University where they were vented to bring the bottles to atmospheric pressure at NAU (0.8 atmospheres). Peptone, phosphate buffer, and vitamin solutions were added anaerobically just before inoculation. Filter-sterilized air was added to each bottle for a final concentration of 1% O_2.

To compare the $^{13}CO_2$ production rate from *T. hugenholtzii* cultures with that of sterile controls, the ideal gas law was used to convert the volume of CO_2 present in the incubations from various processes and additions, to moles of CO_2 present from various processes and additions.

$$PV=nRT$$

Where n is the number of micromoles of gas; P is pressure in atm; V is volume of the gas in L; T is temperature (°K); and R is the gas constant (0.08205746 (atm*L) / (moles*K)). We also used the atom fraction equation for ^{13}C

$$^{13}\text{C atom fraction} = ((\delta 13C/1000+1)*0.011237)/((\delta 13C/1000+1)*0.011237+1)$$

to calculate ^{13}C atom fraction ($x(^{13}C)$) values from $\delta^{13}C$ values determined by running samples on the Picarro. Using the calculated atom fraction values, we applied a mass balance equation for

isotope mixing to determine the contribution of $^{13}C-CO_2$ from different processes and additions in the cultures and abiotic controls.

$$x(^{13}C)_tV_t = x(^{13}C)_1V_1 + x(^{13}C)_2V_2 \ldots$$

Taken together, using cultures with ^{13}C-labeled substrate additions, cultures with no ^{13}C-substrate additions, cultures used for total CO_2 production rates, and abiotic $^{13}C-CO_2$ controls, we were able to attribute $^{13}C-CO_2$ production to either *T. hugenholtzii* metabolism or abiotic processes.

2. Additional information on inferred metabolic potential for *Thermoflexus hugenholtzii* JAD2^T

Inferred amino acid interconversion and biosynthetic capability

Ornithine (M00763) and lysine biosynthesis (M00031) appeared possible (Table S4), yet both biosynthesis pathways were missing genes connecting them to the TCA cycle (Table S3). Histidine degradation to glutamate through N-formiminoglutamate appears possible (M00045) (Table S4). The absence of one gene coding for a homoserine acetyltransferase (EC2.3.1.31) may prevent the synthesis of homocysteine and methionine from aspartate or homoserine (Table S3).

Histidine biosynthesis from ribose-5P was missing a single gene coding for an imidazoleglycerol-phosphate dehydratase (EC4.2.1.19) or a histidinol-phosphatase (EC3.1.3.15) but all other enzymes were present (Table S3). There were no noticeable routes for cysteine biosynthesis by *de novo* means or by conversion of methionine or serine due to the absence of a serine O-acetyltransferase (EC2.3.1.30) (Table S3). *De novo* serine and phosphoserine biosynthesis were not possible due to the absence of a single gene (phosphoserine aminotransferase, EC2.6.1.52) yet, other genes necessary for the conversion of glycolysis-derived glycerate-3P to serine were present (EC1.1.1.95, EC3.1.3.3) (Table S3). The absence of EC2.6.1.52 and genes coding for enzymes for the biosynthesis of aspartate from oxaloacetate suggests an inability for *the de novo* biosynthesis of homoserine, threonine, glycine, and isoleucine (Table S3). Tryptophan biosynthesis from D-erythrose 4-phosphate and

phosphoenolpyruvate appears possible through the shikimate pathway (M00022, M00023) (Table S3, Table S4). Genes coding for enzymes involved in tryptophan metabolism and lysine degradation were largely absent, suggesting these compounds are not important substrates for *T. hugenholtzii* JAD2^T (Table S3). Alanine could be synthesized from or broken down to pyruvate (EC1.4.1.1, EC 2.6.1.44, respectively), but no other pathways for conversion to other amino acids were observed (Table S3). Valine and leucine could be synthesized from pyruvate (TableS3).

Inferred nucleoside/nucleotide biochemistry

T. hugenholtzii JAD2^T appears to be able to degrade and synthesize both adenine and adenosine (Table S3, Table S4). In contrast, neither thymine degradation (M00046) or synthesis (M00053) appear to be possible due to the absence of multiple enzymes (EC2.4.2.6, EC2.4.2.4, EC1.3.1.1, EC1.3.1.2, EC1.17.99.4) (Table S3, Table S4).

3. Additional information for exometabolomics

Accumulation and degradation of other compounds

Adenine and adenosine were substrates for *T. hugenholtzii* JAD2^T. This observation was supported by the presence of nucleoside transporters and complete pathways for the degradation of these compounds. Biosynthetic pathways for this compound were also complete in *T. hugenholtzii* and all MAGs, indicating *Thermoflexus* may synthesize them *de novo* when necessary. In contrast, thymine accumulated in the medium, which seems paradoxical given that thymine pathways are incomplete. This was unexpected given that biosynthesis pathways for this compound are incomplete in *T. hugenholtzii* JAD2^T and all *Thermoflexus* MAGs.

A slight increase in riboflavin in the presence of growth was observed in the exometabolomic data despite a gene coding for an ECF-type riboflavin transporter, S component being found. More significant is the thermal degradation of riboflavin, which is thought to be stable at higher temperatures over short periods of time. The long incubation times necessary to grow *T. hugenholtzii* JAD2^T provide ample opportunity for thermal degradation and production of

products, providing deviations in the chemical makeup of the medium over time solely due to temperature. This, along with the demonstrated production and degradation of other compounds due to temperature, highlights the importance of running sterile controls in parallel with cultures during exometabolomic analyses but also illuminates potential challenges when growing thermophiles with long doubling times, due to chemical changes in the medium strictly from temperature.

4. Cell yields for exometabolomics and an unidentified GBS organic extract

An unidentified GBS organic extract stimulated Thermoflexus growth

The addition of an organic extract derived from Great Boiling Spring, the source of *T. hugenholtzii* JAD2^T, significantly enhanced growth, suggesting that organic extracts commonly used for microbiological media are limited in some beneficial nutrients. A thiamine transport system was found in *T. hugenholtzii* JAD2^T, and an ascorbate phosphotransferase system was conserved across the genus, which is consistent with our observations that *T. hugenholtzii* JAD2^T is stimulated by high concentrations of vitamins (see below). Complete carbohydrate metabolic pathways and a variety of transporters for oligo- and monosaccharides suggest these compounds may be utilized by *T. hugenholtzii* JAD2^T and other *Thermoflexus* sp., yet these types of compounds are not capable of serving as sole carbon and energy sources for growth (Dodsworth et al., 2014). These data suggest that yet-to-be-determined key nutrient limitations may contribute to the low cell density observed in *T. hugenholtzii* JAD2^T cultures when grown on peptide-based complex media as a carbon and energy source.

Preparation of Hot Spring Organic Extract

Bulk spring water was collected from GBS by tangential flow filtration and stored in sterile plastic carboys at room temperature until processed. GBS water was flowed through Diaion HP-20 (Supelco Analytical) at ~150 mL per min by a peristaltic pump. Approximately 40 L of hot spring water was passed through the resin. The resin was then rinsed with nanopure water to remove residual salts, and 60% acetonitrile was used to collect the organic extract. The organic extract was dried in 2 mL Eppendorf microtubes using a Speed Vac SC100 (Savant Instruments, Inc., Farmingdale, NY, Model: RT100A) on medium drying rate. The dried extract was solubilized in anaerobic nanopure water in an anaerobic chamber at a concentration of 800 µg/mL. The organic

extract was filter-sterilized using a 0.2 µm filter (VWR, N.A. PN: 28145-501) and was stored anaerobically at 4°C in the dark. Growth experiments using the organic extract were carried out as described above for exometabolomics but with the addition of 32 µg/mL (final concentration) to the base medium, these results were not analyzed in this manuscript. Attempts at chemically describing the organic extract using HPLC-MS/MS failed.

Cell counts for exometabolomic experiments and hot spring organic extract additions. Replicate 1 for exometabolomics demonstrated much higher growth than the other replicates within the same treatment.

	cells/mL	Average (cells/mL)
Exometabolomics		
Rep. 1	4.6×10^7	1.1×10^7
Rep. 2	2.7×10^6	
Rep. 3	2.0×10^6	
Rep. 4	1.4×10^6	
Rep. 5	2.6×10^6	
Organic extract addition		
Rep. 1	5.1×10^7	3.6×10^7
Rep. 2	3.5×10^7	
Rep. 3	1.8×10^7	
Rep. 4	4.7×10^7	
Rep. 5	3.2×10^7	
